# Modelling the Evolution and Spread of HIV Immune Escape Mutants

**DOI:** 10.1371/journal.ppat.1001196

**Published:** 2010-11-18

**Authors:** Helen R. Fryer, John Frater, Anna Duda, Mick G. Roberts, The SPARTAC Trial Investigators, Rodney E. Phillips, Angela R. McLean

**Affiliations:** 1 The Institute for Emerging Infections, The James Martin 21st Century School, Department of Zoology, Oxford University, Oxford, United Kingdom; 2 The Institute for Emerging Infections, The James Martin 21st Century School, The Peter Medawar Building for Pathogen Research, Nuffield Department of Clinical Medicine, Oxford University, Oxford, United Kingdom; 3 Institute of Information and Mathematical Sciences and New Zealand Institute for Advanced Study, Massey University, Auckland, New Zealand; University of Texas at Austin, United States of America

## Abstract

During infection with human immunodeficiency virus (HIV), immune pressure from cytotoxic T-lymphocytes (CTLs) selects for viral mutants that confer escape from CTL recognition. These escape variants can be transmitted between individuals where, depending upon their cost to viral fitness and the CTL responses made by the recipient, they may revert. The rates of within-host evolution and their concordant impact upon the rate of spread of escape mutants at the population level are uncertain. Here we present a mathematical model of within-host evolution of escape mutants, transmission of these variants between hosts and subsequent reversion in new hosts. The model is an extension of the well-known SI model of disease transmission and includes three further parameters that describe host immunogenetic heterogeneity and rates of within host viral evolution. We use the model to explain why some escape mutants appear to have stable prevalence whilst others are spreading through the population. Further, we use it to compare diverse datasets on CTL escape, highlighting where different sources agree or disagree on within-host evolutionary rates. The several dozen CTL epitopes we survey from HIV-1 gag, RT and nef reveal a relatively sedate rate of evolution with average rates of escape measured in years and reversion in decades. For many epitopes in HIV, occasional rapid within-host evolution is not reflected in fast evolution at the population level.

## Author Summary

HIV evolves so quickly that it can be seen to adapt within one infected person. Evolutionary escape from immunity is particularly well-described. Escape variants transmit to new hosts, where they may revert. We present a mathematical model of three processes: within-host evolution of escape mutants, transmission of those variants between hosts and subsequent reversion in new hosts. Using this model we reconcile diverse datasets on HIV immune escape, highlighting where multiple data sources agree or disagree on the underlying rate processes. The several-dozen immune epitopes we survey reveal a relatively sedate rate of evolution with average rates of escape measured in years and reversion in decades. Although there are frequent reports in the literature of early and rapid within-host evolution of HIV, for many epitopes this is not reflected in fast evolution at the population level.

## Introduction

During the course of a single infection HIV evolves to escape from the selection pressures imposed by its host's immune response. Such changes have been recorded under selection from all three arms of the specific immune response, but escape from CD8+ cytotoxic T lymphocytes is particularly well documented [1], [2], [3], [4], [5]. HIV variants that cannot be recognised by current host CTLs are termed “CTL escape mutants”. Such mutants have been shown to transmit from one host to another [6], [7], raising their status from potential causes of pathogenesis within individuals [8], [9], [10], [11] to potential drivers of evolutionary change across the global HIV pandemic [12], [13], [14], [15], [16].

Different hosts make immune responses to different parts of HIV (known as epitopes) and for CTL responses the epitopes that can be recognised are determined by the host's class 1 human leukocyte antigen (HLA) type. CTL escape mutants can revert to the wild-type when they are no longer under selection pressure from host immune responses [17], [18]. Global change in HIV's CTL antigens is therefore driven by three parallel processes: the selection of escape mutants in hosts whose immune response can recognise a given epitope (HLA matched hosts), transmission to new hosts, and reversion of escape mutants in hosts unable to recognise the epitope in question (HLA mismatched hosts).

A large literature describes the evolution of HIV CTL escape mutants within individual hosts. Many of those papers are case reports of the timing and speed of outgrowth of escape mutations within an individual and in most cases the events described occur during the first year of infection [3], [5], [19], [20]. The accumulated wisdom from this literature is that the evolution of HIV is always very rapid, that this is strong evidence that CTL immune responses are highly effective and that this viral evolution would pose a severe threat to the durability of any HIV vaccine. This is a received wisdom that is worth serious review as it has profound influence on how we think about the interaction between HIV and its human hosts.

A better understanding of the global tempo of antigenic change in HIV can be achieved by addressing a series of specific questions. On average, how fast do HIV escape mutations arise in HLA matched individuals? How fast do reversions occur in HLA mismatched people? HIV is a relatively recently emerged infection of humans; so is it still adapting to its new hosts, and if so, how fast? What is the relationship between the tempo of adaptation within individuals and the rate of genetic change across the entire pandemic? If HIV is still adapting, what patterns can we expect to unfold across the population of infected people? How will those patterns be different in people of different HLA types and in populations with different HLA frequencies?

Some of these questions have been elegantly addressed in large observational studies which describe the patterns of events that have unfolded in recent decades [12]. In order to understand the processes that underlie those patterns, and to predict what future patterns we might expect we need mathematical models of within-host evolution and between-host transmission that are firmly rooted in the relevant data. There is a substantial literature on mathematical models of the evolution of HIV. Much of it has focussed on the within-host dynamics of HIV variants, either selected by immune responses [4], [21], [22] or by antiviral drugs [23], [24], [25]. Another literature focuses on models of the spread of drug resistance [26], [27]. By comparison, limited attention has been paid to the two-level problem of the evolution of CTL escape mutants within hosts and the spread of those mutants between hosts [28].

## Results

**Figure 1:**
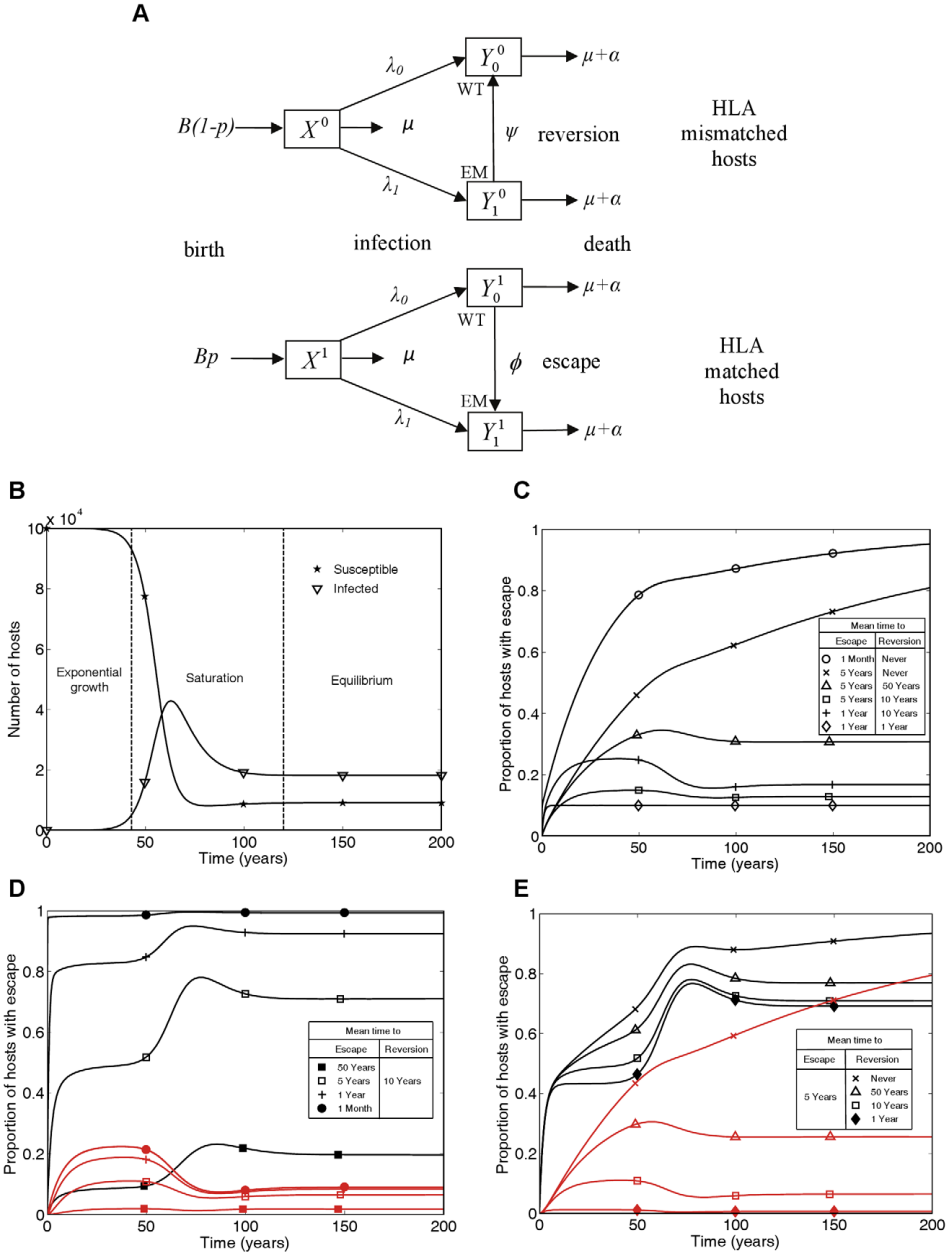
A mathematical model of within-host evolution and between-host transmission of escape mutants at a single CTL epitope. A) The mathematical model in schematic form, where WT and EM denote the wild-type strain and escape mutant strain, respectively. B) Changes in numbers of susceptible (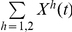) and infected (
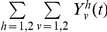
) hosts over time showing the three phases of the epidemic: exponential growth, saturation and equilibrium. C) Changes through time in the proportion of infected hosts with escape at a single CTL epitope for different escape and reversion rates. D and E) Changes through time in the proportion of HLA matched (black lines) and HLA mismatched (red lines) hosts infected with escape at a single CTL epitope for different escape and reversion rates. Different escape rates are compared in D) and different reversion rates are compared in E). The following initial conditions and parameters were used for these plots: *X^1^*(0) = 10^4^, *X^0^*(0) = 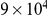, 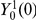 = 0.1, 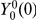 = 0.9, 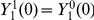 = 0, *p* = 0.1, *μ* = 1/50 years^−1^, *μ*+*α* = 1/10 years^−1^, *β*c = 0.3 and *B* = 10^5^ *μ* years^−1^. These parameters yield a basic reproduction number of 3, since for this model *R*_0_ = *β*c/(*μ*+*α*).

In order to address this gap we have developed a mathematical model that simultaneously captures events while viruses evolve within individuals and tracks the spread of variants as viruses are transmitted between individuals. Between-host transmission is modelled using a standard mathematical description of the frequency-dependent transmission of an infectious disease from which there is no recovery – the so-called SI model. However, the model we present allows host-heterogeneity with respect to a single HLA type so that some hosts have the potential to make a CTL response to a given viral epitope, whereas other hosts do not. Viral evolution is captured by allowing viral heterogeneity with respect to the presence – or not – of escape mutations in a single epitope restricted by the host HLA under consideration. Thus there are four types of infected hosts: HLA matched hosts infected with wild-type or escape mutant virus, and HLA mismatched hosts infected with wild-type or escape mutant virus. Thus there are no mixed infections, or more precisely each host can only be infectious with one type of virus. Only HLA matched hosts infected with wild-type virus can mount effective CTL responses to the viral epitope under consideration. They drive the evolution of CTL escape mutants and can therefore switch to become HLA matched hosts infected with escape mutant virus. HLA mismatched hosts are unable to mount CTL responses to the given epitope whatever mutations it bears and they can therefore allow their infecting virus to revert from escape mutant to wild-type. In this model such viral reversion is represented by HLA mismatched hosts switching from being infected with the escape mutant virus to being infected with the wild-type virus. Every infected host is infectious with the viral type they carry, so that the two viral types are transmitted between individuals at rates driven by the proportion of the total population infected with each. A diagram of the model is presented in Figure 1A and the parameters, variables and equations defining the model are provided in the Methods section. Figure 1B illustrates the three phases of an epidemic predicted by our model (and therefore also by the standard SI model): initial exponential growth, saturation, and then stabilisation at an endemic equilibrium. The model structure and parameter values we have used are appropriate for modelling HIV within a single, closed high-risk group. Since the majority of the data that we analyse will be from individuals belonging to high risk groups this is an appropriate approximation.

This model has similarities to mathematical models of the spread of drug resistance [29]. HLA matched hosts being equivalent to hosts taking antiviral drugs and CTL escape mutant virus equivalent to drug resistant virus. However, in this model, hosts never change their type so the model is structurally different from drug resistance models and new analyses are needed.. Unlike the gene-for-gene models of the world of plant pathology [30] this model is also not designed to consider host and pathogen co-evolution. Here, birth rates are independent of current host densities (see Figure 1A) so a different model structure that combined pathogen evolution as explored here and host changes as explored, for example, in Cromer et al. (2010) [31], would be needed to explore co-evolution of HIV and humans.

Figure 1C summarises the time-course of population prevalence for an escape mutant that arises at the start of an epidemic. Three features are noteworthy. First, there are qualitatively different patterns for mutants with different rates of escape and reversion. For escape mutants that never revert or revert very rarely (e.g. once in 50 person-years of observation) we would currently expect the prevalence of escape in the population to be increasing. On the other hand, if reversion is more rapid, we would expect the population prevalence of escape to have already stabilised at a plateau before entering a second transient phase to reach its eventual equilibrium value. The early plateau occurs because at this stage, although the number of HLA matched and HLA mismatched hosts with each virus type are growing exponentially, they are growing at equal rates. Second, the predicted prevalence of escape is both qualitatively and quantitatively very sensitive to the reversion rate if reversion is slow. Notice the dramatic difference in the long-term between a zero rate of reversion and a very slow rate of reversion (Figure 1C crosses versus triangles). If reversion never happens then the escape mutation will eventually fix – although this could take centuries. However, even a rate of reversion that could only be observed in a large cohort study (once in 50 person-years of observation) would prevent fixation of the escape mutant in the population, with an initial rise in prevalence followed by a fall and eventual stabilisation. Thirdly, faster rates of escape and slower rates of reversion lead to higher population prevalence of escape. However the underlying epidemic dynamics of in the community under study and the proportion of HLA matched hosts in the population [12] will also affect the prevalence of escape.

In each of Figures 1D and 1E escape prevalence is tracked in the two different host populations: HLA matched (black lines) and HLA mismatched (red lines). As one would expect, escape prevalence is always higher in HLA matched than HLA mismatched hosts and increases with faster escape rates (Figure 1D) or slower reversion rates (Figure 1E). Furthermore, when reversion takes an average of approximately 10 years or less, the prevalence in both host types achieves a temporary plateau during the exponential phase of an epidemic. Analytic expressions for the temporary plateau, the long-term equilibria and the time-course of escape prevalence during the initial years of the epidemic are presented in the Text S1.

In Figure 1 we plot results as prevalence of infection with escape mutant viruses in infected hosts of different types. Incidence is also of interest, but in this model incidence is driven by prevalence so the proportion of new infections that carry escape mutations will always be the same as the proportion of prevalent mutations that carry escape mutations.

**Figure 2:**
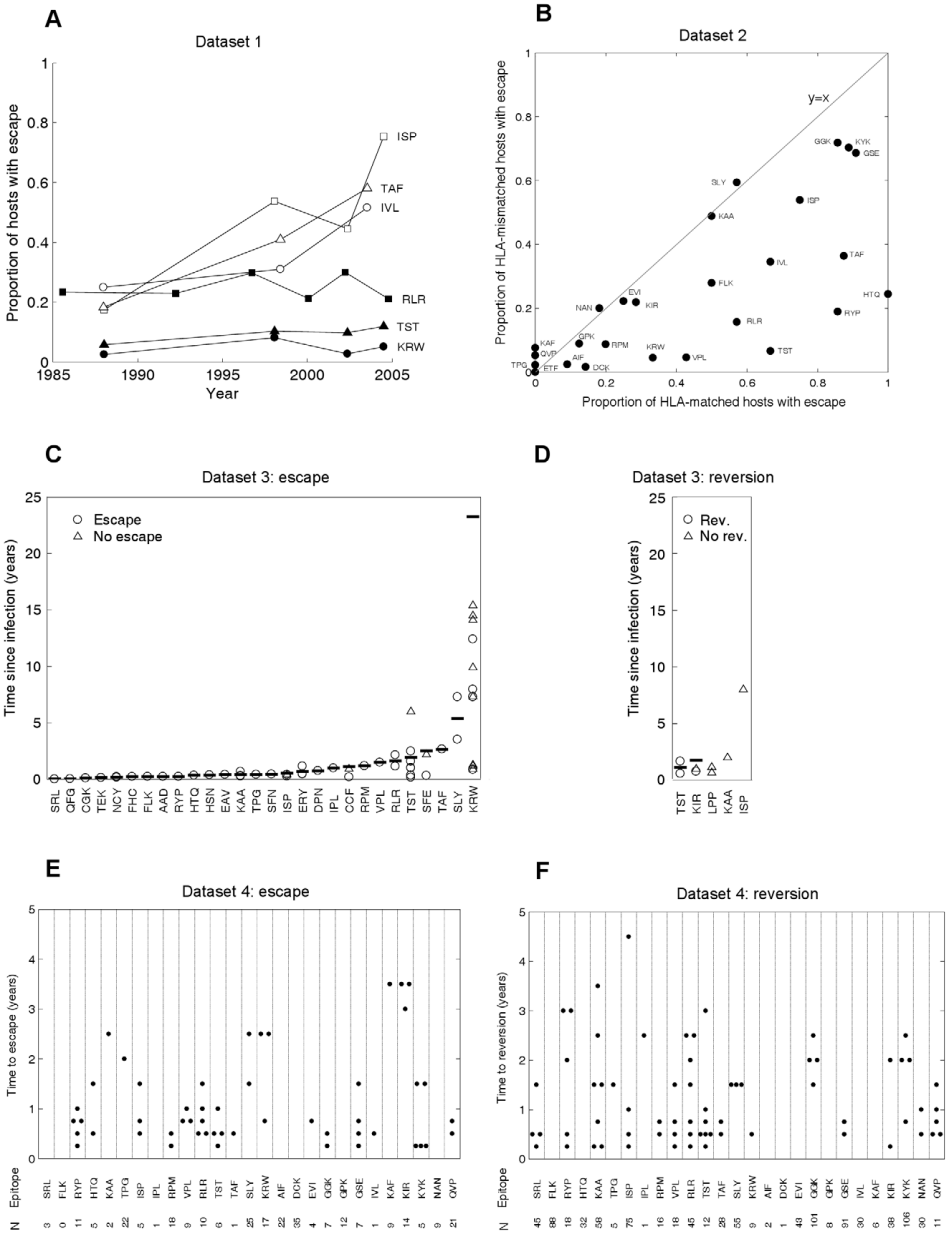
CTL escape and reversion data from the current HIV pandemic. A) Dataset 1: the evolution of previously described escape mutants in six CTL epitopes. Data from dated B-clade sequences provided in the Los Alamos database. The filled shapes show three epitopes for which the proportion of hosts with escape has remained relatively invariant over the past 20 years. The unfilled shapes show three epitopes for which the proportion of hosts with escape has increased over the last 20 years. B) Dataset 2: cross sectional data describing the proportion of HLA matched and HLA mismatched hosts with described escape mutants in gag, RT and nef. Each dot represents the data for a single CTL epitope (N = 26). Data from 84 chronically infected hosts from Switzerland. C) Dataset 3: escape data from individual case reports described in the literature. Each marker represents the results from one HLA matched host infected with the wild-type epitope at the first sample time. In cases where escape occurred the time between infection and escape is represented by a circle. In cases where escape did not occur the time between infection and the last sample is represented by a triangle. The inferred average time to escape is represented by a horizontal bar. These averages account for data, where available (triangles), from hosts in whom escape mutants do not appear (see Table S3 for details). D) Dataset 3: reversion data from individual case reports described in the literature. Each marker represents the results from one HLA mismatched host infected with a described escape mutant at a particular epitope at the first sample time. The markers are analogous to those described for C). E) Dataset 4: escape data from a longitudinal cohort of 189 acute seroconverters. Estimates are provided for 27 epitopes with previously described escape mutations in gag, RT and nef. These are largely the same epitopes shown in B), though there is some lack of overlap due to the absence of certain data from one or other dataset. N is the number of HLA matched hosts infected with the wild-type epitope at the first sample. In cases where escape occurred, the time between infection and escape is represented by a dot. F) Dataset 4: reversion data from the same longitudinal cohort of individuals. For each epitope N is the number of HLA mismatched hosts infected with an escape mutant at the first sample. In cases where reversion occurred, the time between infection and reversion is represented by a dot.

The model's behaviour can be compared with CTL escape data from the current HIV pandemic. Such data are available from diverse studies, summarised in Figure 2. Throughout this study we define escape as *any* mutation at a site at which an escape mutation has been described (and phenotypically demonstrated *in vitro*). Escape data are available at two levels of organisation: comparisons across individuals (Figures 2A and 2B) and changes within individuals (Figures 2C–F). Figure 2A (dataset 1) tracks changes through time in the proportion of hosts with escape mutations in six different epitopes. These data were downloaded from the Los Alamos HIV sequence database (www.hiv.lanl.gov) using a search for dated B-clade sequences and eliminating duplicate samples from the same individual. Although this database is not strictly an epidemiological survey, it is the largest source of temporal population level data. The six epitopes are a subset of 31 epitopes in gag, reverse transcriptase (RT), and nef for which at least one escape mutation has been described in the literature. Details and references for these mutations are provided in Table S1 and Text S2. None lie at defined drug resistant sites according to Stanford HIV Drug Resistance Database (http://hivdb.stanford.edu/). As predicted in Figure 1C, different epitopes show different behaviour; in some the prevalence of escape has remained stable over several decades (filled markers), whereas in others the prevalence of escape has been increasing (unfilled markers). In Figure 2B (dataset 2), for 26 epitopes with described escape mutants in gag, RT and nef the prevalence of escape in HLA matched and HLA mismatched hosts amongst 84 individuals with chronic infection is presented. These patients, the majority (76%) of whom have HIV-1 subtype B, have been described in detail elsewhere [32], [33], [34]. Data for the remaining 5 (out of 31) epitopes were not available (Table S2). The data presented in Figure 2B should be compared to the model predictions in Figures 1D and 1E. As expected, escape is more prevalent in HLA matched than mismatched individuals (for given parameters black lines are above red lines in 1D and 1E, while points in 2B lie below the line y = x), but otherwise, across different epitopes the prevalence of escape is very variable (at a fixed time point in 1D or 1E prevalence of escape for different epitopes can vary widely, in 2B epitopes are liberally scattered across the bottom right half). The other type of data on the dynamics of escape and reversion tracks events as they occur within infected individuals. One source of such data (dataset 3) is case reports of single HIV-1 infections, recording the time after infection when escape or reversion occurred. Around 55 such escape events in 28 different epitopes across the full genome are described in the literature (Figure 2C, Table S3 and Text S2), but only 3 such reversions (Figure 2D, Table S4 and Text S2). For most epitopes there are only one or two records of time to escape and those are within the first few years of infection. It is common for the data summarised in 2C to be taken as indicating that the rate of escape is generally rapid. Collating individual records of time to escape cannot yield an estimate of the rate of escape as these studies typically ignore the existence of individuals in whom nothing interesting happens. To estimate escape and reversion rates longitudinal cohort studies are typically used. Early results from one such cohort study of 189, acute seroconverters are summarised in Figures 2E and 2F (dataset 4). These individuals are mostly B-clade (87%) and have been described previously [35]. They were first sampled a median of 6 weeks following their estimated date of seroconversion and were followed for a mean further 1.9 years (range: 0.5–5 years). It is clear from Figure 2E that the published literature on time to escape (Figure 2C) is heavily biased towards early escape events and that when a cohort is followed, amongst all hosts who are HLA matched for any given epitope and infected with wild-type at first sample (N in Figure 2E), many show no escape in the early years of infection. The absence of escape events amongst many hosts implies that escape is slow. For example, the average time to escape in epitope KRWIILGLNK (HLA B27-restricted, HXB2 p24 gag 131–140) is 11.1 years because escape events occurred in only 3 out of the 17 HLA B27 hosts who had the wild-type at the first sample. Reversion events are similarly sparse in comparison to the numbers of HLA mismatched hosts who had each escape mutant at first sample (Figure 2F). Reversion rates are therefore also slow.

We do not have to wait several decades for longitudinal cohort studies to play out. Our model can be used to infer rates of escape and reversion from HLA-typed escape prevalence data such as that shown in Figure 2B. To make these inferences we need estimates of the basic reproduction number, *R*_0_, (defined as the expected number of secondary cases arising from a typical infected individual when all other members of the community are susceptible), the average life expectancy of infected hosts, the duration of the HIV epidemic in the sample population and the proportion of the population who are HLA matched for each epitope. Since the data in 2B are from Switzerland, we assume that HLA prevalences are equal to those found in Caucasians [36] and that the epidemic duration at the time of sampling (year 2000) is 27 years [37]. Further, we use a basic reproductive number of 3 [38] and an average life expectancy of infected hosts of 10 years [39]. With these parameters fixed, we use the model to fit only two parameters – the escape and reversion rates – from two observations – the proportions of HLA matched and HLA mismatched hosts with escape. For fixed model parameters, escape prevalences in both host types strictly increase with faster escape rates and slower reversion rates, thus any unique pair of rates correspond to a unique pair of escape prevalences. The model can therefore be fitted very simply using ‘least-squares’ to find the unique pair of escape and reversion rates that minimise the difference between the observed and expected escape prevalences.

**Figure 3:**
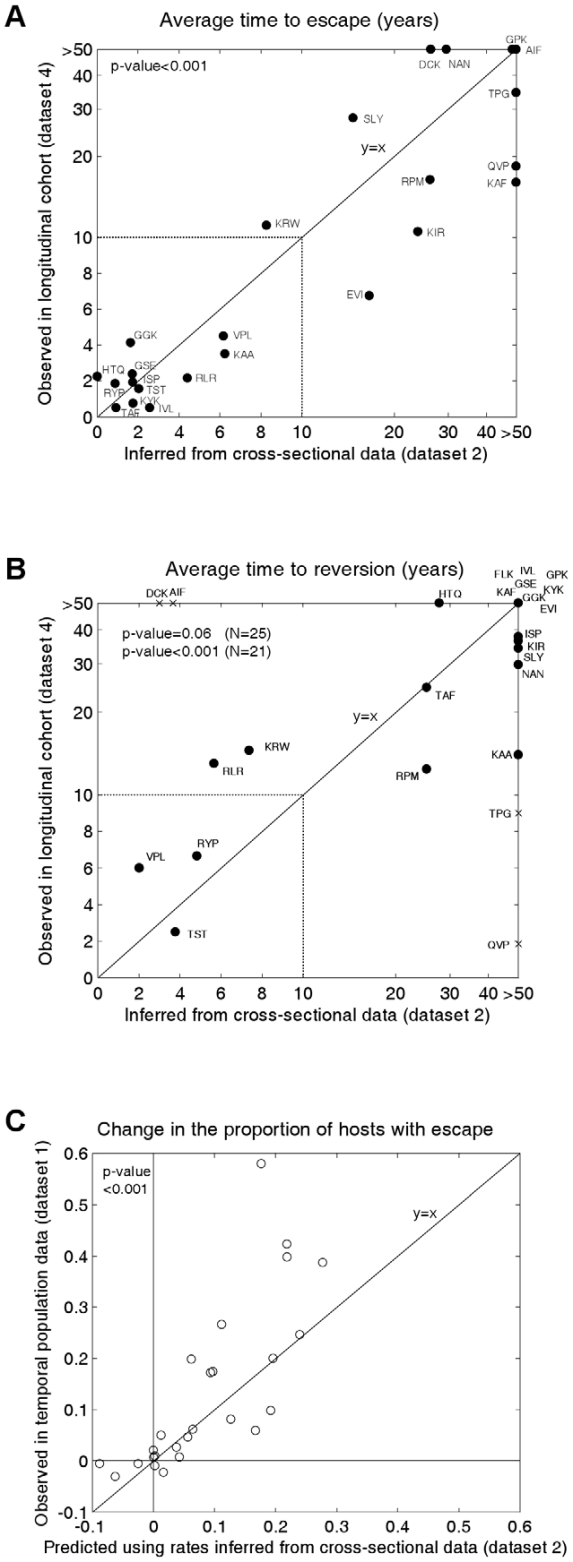
Observed and inferred escape rates, reversion rates and changes in escape prevalence. A) A comparison of the mean times to escape inferred from dataset 2, the cross-sectional data (x-axis) and observed in dataset 4 (Figure 2E), the longitudinal cohort study (y-axis). B) A comparison of the mean times to reversion inferred from dataset 2 and observed in dataset 4 (Figure 2F). For A) and B) estimates are provided for epitopes in gag, RT and nef for which escape mutants have been described and for which data are available from both studies. The inferred rates (x-axes) are calculated from dataset 2 using the mathematical model. For A) the observed times to escape (y–axis) are calculated from dataset 4 by considering all HLA-matched hosts who have the wild-type epitope at the first sample. We then sum over all person-years of observation for which an escape mutant is absent and divide by the number of hosts in whom escape mutants emerge. For B) reversion rates from dataset 4 are estimated using an analogous method from all HLA-mismatched hosts who have an escape mutant at the first sample. Note that the epitopes in these graphs are the same as those presented in Figure 2B, except that, epitope FLK is absent from A) and epitope ETF is absent from both A) and B) because the relevant estimates were not available from dataset 4. The data are presented on a linear scale from 0–10 years and on a log scale beyond 10 years. In B) the crosses represent the four epitopes for which we have the least confidence in our inferred reversion rates (see Figure S1). The remaining epitopes are shown as circles. C) A correlation between observed and predicted changes in the escape prevalence of described escape mutants in gag, RT and nef in the population between approximately 1995 and 2005. These are the same 26 epitopes as shown in dataset 2. The observed changes are from sequence data downloaded from the Los Alamos Database (dataset 1, Figures 2A and S3). The predicted changes over the same period are estimated using the mathematical model parameterised by the escape and reversion rates inferred from dataset 2 (x-axes 2A and 2B).

Figure 3 shows the inferred mean time to escape (3A; x-axis values) and mean time to reversion (3B; x-axis values) for each epitope estimated from the cross-sectional escape prevalence data in Figure 2B (dataset 2). Ninety five percent confidence intervals surrounding these estimates are shown in Figure S1. Confidence differs between epitopes because of differences in the underlying evolutionary rates and in the number of hosts who are HLA matched and mismatched for each HLA type. Nevertheless, even with our relatively small total sample size (84 individuals), for most epitopes the confidence is sufficient to distinguish between rates measured in months, years or decades. These confidence intervals account for sampling errors, but assume that the structure and parameters of the model are a perfect representation of the system. Figure S2 shows how our assumed global parameters (the basic reproductive number, life expectancy of infected hosts, epidemic duration and HLA prevalences) affect our inferences. These analyses reveal that while the magnitude of our escape and reversion rates change with each of these parameters, their rank orders remain largely preserved.

To test our model predictions, escape and reversion rates for the same epitopes were determined from the independent longitudinal cohort data (dataset 4) presented in Figures 2E and 2F. Although this cohort is relatively new it is still possible to estimate mean escape and reversion rates by taking account of all individuals, those who demonstrate escape or reversion and those who do not. Since very many individuals have not yet demonstrated escape or reversion (few dots in Figures 2E and 2F compares to N for each epitope) many of our estimates of mean escape and reversion rates are long compared with the duration of the cohort study. Thus the fact that the y–axis of Figure 3A runs to >50 years whilst that of Figure 2E only extends to 5 years is a reflection of the large number of person-years of observation in which no escape occurs summarised in Figure 2E. Figure 3A reveals that the inferred escape rates estimated from the two population studies are not just highly correlated (Pearson's correlation coefficient (PCC) = 0.83, 2-tailed p-value<0.001, N = 24) but are also approximately equal in magnitude (2-tailed paired t-test, H0: difference = 0, t-value = −0.17). Figure 3B also shows a positive correlation between the observed and inferred reversion rates. For this comparison significance just misses the standard 0.05 p-value boundary (Kendall Tau correlation coefficient (KTCC) = 0.32, p-value = 0.06, N = 25). It is noteworthy, however, that the correlation is highly significant (KTCC = 0.68, p-value<0.001, N = 21) if we exclude the four epitopes for which we have least confidence in our inferred rates (Figure S1). These reversion rates are also approximately equal in magnitude (2-tailed paired t-test t-value = 0.87, N = 21).

We can take the inferred rates of escape and reversion and use the model to calculate a predicted change over 20 years (1985–2005) in the population escape prevalence of each epitope. When we compare that predicted change with the observed change in sequences deposited in the Los Alamos database (Figures 3A, dataset 1, Figures 2A and S3) we find that these values are strongly correlated (Figure 3C; PCC = 0.78, 2-tailed p-value<0.001, N = 26) and not significantly different from each other (2-tailed paired t-test t-value = −1.58). Thus, the model is able to explain why some escape mutants appear to have stable prevalence, whilst others are spreading through the population. To calculate these predicted changes, the initial conditions of the model are defined so that the proportion of hosts with escape is equal to the proportion observed in dataset 1 at the earliest sample time. Since individuals in dataset 1 are not HLA-typed, the initial conditions are also defined so that the ratio of HLA matched to HLA mismatched hosts with escape is equal to the ratio observed in the cross sectional data (Figure 2B).

Our interpretation of the data in Figure 2 is that datasets 1, 2 and 4 yield consistent estimates across many epitopes. Both escape and reversion rates are slow. It is only the data from small studies of one or two people in dataset 3 which imply that escape rates are usually rapid. The studies summarised in 2C are mostly individual case reports and do not give a reliable picture of time to escape as there is strong publication bias in favour of papers describing escape events and against case studies of individuals in whom nothing happened. Escape rates estimated from population data, whether longitudinal (Figure 2E and y axis Figure 3A) or cross-sectional (Figure 2B and x axis Figure 3A) reveal that, on average, escape is typically much slower than the individual case reports suggest. As shown in Figure 2C, escape has previously been described as typically occurring within the first year of infection (median time to escape = 0.44 years; interquartile range (IQR) = 0.25–1.3 years); however, our population-based estimates imply that only 3 out of the 26 epitopes surveyed here have an inferred mean time to escape of less than a year. Across all 26 epitopes the median inferred time to escape is 8.0 years and the IQR is 1.8–34.0 years. It must be emphasized that these estimates refer to escape in any HLA matched hosts. Hosts who are HLA matched for a given epitope have the potential to make an immune response to that epitope, but do not necessarily do so. Indeed, one study indicates that, on average, responses to any given epitope are made by only a third of HLA matched hosts [33]. Escape rates in the presence of an immune response could therefore be three times faster than the rates estimated in this study and this may go part way towards explaining why the escape rates derived from the case-reports – where CTL responses are measurable – are faster than those estimated here. However it is not enough to explain the close-to 20-fold difference uncovered here. Our assumed global parameters also do not explain this difference. Plausible alternative parameter choices do change the magnitude of our inferred rates, but not to this extent (Figure S2).

Our inferred reversion rates are even slower than our inferred escape rates: there is no reversion in 56% of epitopes and at the lower quartile the average time to reversion is 6.5 years. As shown in Figure 1C, however, even a slow but non-zero reversion rate can prevent fixation of an escape mutant at the population level. The longitudinal cohort also reveals that reversion is slow (time to reversion: median = 36.4 years, IQR = 13.0 years-no reversion). For neither dataset do the escape rates correlate with the reversion rates.

It is surprising to find that the rate of escape from CTL selection is more than an order of magnitude slower than suggested in a substantial literature describing events in carefully followed individuals. It is reassuring that the result arises very consistently from two different analyses of two independent datasets. Estimating escape and reversion rates from longitudinal cohort data is a straightforward and well-established process (see Figure 3 legend). Our estimates from the cross-sectional data require inference based on the new model we have presented here. That model is deliberately kept simple to allow a transparent explanation of what assumptions we have made and to minimise the number of other parameters we must fix when using the model to estimate escape and reversion rates. But with such a simple model the question must arise, is the result simply an artefact of leaving out too much of the relevant biology? In what follows we explore a series of six complicating factors that might change the rates of escape and reversion inferred from the cross sectional data. In each case we find that our results are robust to the inclusion of extra biological complexities in our model structure or alternative definitions of escape mutants. The details of data interpretation and model development are, for the sake of brevity, presented in the supplementary materials. Here we summarise the results.

Might our definition of escape have excluded many genuine escape mutations? We confined our analysis to mutations at sites that have been demonstrated *in vitro* to confer escape. This curbs both the epitopes we look at and the sites within those epitopes that we consider. To check for bias arising from only looking at sites with defined escape we replot Figure 2B redefining escape as any mutation within the epitope (Text S3, factor 1). We find that, for all but 2 of the 26 epitopes, any increase in the inferred escape rate would be marginal or none. To check for bias arising from our choice of epitopes we replot Figure 2B to include all known epitopes in gag, RT and nef, regardless of whether or not an escape mutant has ever been described in that epitope. The figure includes the 26 epitopes we have already investigated and 48 other epitopes for which no escape mutation has ever been described. All of the 48 new epitopes have roughly equal prevalence of mutated epitopes in HLA matched and mismatched hosts. Thus, if anything, our choice of epitopes has tended to focus attention on those epitopes where escape and reversion are faster.

It is an inbuilt assumption of our model that escape mutants do not revert in HLA matched hosts. In principal such reversion could lead to lower prevalence of escape in HLA matched hosts and consequent underestimation of escape rates. The longitudinal cohort study (dataset 4) allows an estimate of the rate at which escape mutants revert in HLA matched hosts. The estimate is that this occurs, on average, once every 25 person-years of observation. Using this estimate and a modified version of our model in which escape mutants can revert in HLA matched hosts we can re-estimate escape and reversion rates from the data in Figure 2B and the new model (Text S3, factor 2). We find that the new estimates of escape rates are marginally (less than 10%) faster and that reversion rates are barely affected at all. If the rate of reversion in HLA matched hosts is as small as that observed in our longitudinal study then such reversion would not substantially affect inferred rates of escape and reversion.

Another possibility is that some escape mutations only appear transiently, to be replaced by other mutations in the same epitope. We develop a model of such a process in Text S3 (factor 3). We find that transitions between different escape mutants at the same epitope would not affect the evolution of escape mutants at the population level. This is because if an individual with one particular escape mutant selects another escape mutant in place of the first, the total number of hosts with escape remains the same. As a result, transitions between escape mutants would not affect the rates of escape and reversion inferred from the cross-sectional data using the original model.

We have assumed that the rates of escape and reversion are homogenous across the duration of infection. In principal, if escape is faster earlier on during infection the prevalence of escape mutants in the population would be lower than would be predicted under the assumption that escape is as fast late on as it is early on during infection. This would lead us to underestimate the rate of escape during the early stage of infection. Instead, our estimate would represent a form of average escape rate across both the early and late stages of infection. Likewise, faster reversion in early infection could affect the prevalence of escape mutants and thus our inferred rates. Using a model in which escape and reversion are both faster in the first year of infection and an estimate (based on an upper bound from the longitudinal cohort study) that both rates halve after the first year we re-analysed the cross-sectional data to see how estimates change under these different assumption. We found that both inferred escape and reversion rates are faster under these new assumption but the halving of rates after 1 year is not large enough to have a substantial effect upon the escape prevalence at the population level and thus upon our inferred rates of escape and reversion. Details of the model and of the data supporting a halving of rates after one year are presented in Text S3 (factor 4).

We have also assumed that people are equally infectious throughout their infection. A study of HIV-discordant heterosexual couples [40] found that transmission is 10 times more likely in the first 2.5 months of infection compared with the chronic phase of infection. We developed a model in which transmission is much faster during acute infection than later (Text S3, factor 5). We found that realistic differential transmission rates between acute and chronic infection would have very little impact upon the prevalence of escape mutants in the population. There are two reasons for this. Firstly, even if, as estimated, transmission is 10 times faster during acute infection than during chronic infection, acutely infected hosts still account for a minority of infections because acute infection is short compared to the whole duration of an infection. Indeed, it is estimated that only 15–20% of all infections can be attributed to acutely infected hosts [40], [41]. In addition to this, the escape prevalence at the population level is highly dependant upon the rates of within-host evolution and not just upon the transmission of escape mutants between hosts. Model simulations show that together these factors would have no noticeable impact upon the evolution of escape mutants or upon our inferred rates of escape or reversion.

Throughout this analysis we have treated different epitopes as though they were independent entities. Of course that is not so and the most intimate way in which epitopes can interact is by lying in identical parts of the HIV genome. Many CTL epitopes do lie in overlapping sections of the genome and we therefore developed a model to investigate the dynamics of a mutation at a single site that confers resistance in two overlapping epitopes restricted by two different HLA alleles (Text S3, factor 6). We find that analysing overlapping epitopes as though they were independent of each other typically leads to underestimation of the reversion rate but overestimation of the escape rate. Overlapping epitopes therefore cannot explain why the escape rates estimated from the cross-sectional data are so slow.

## Discussion

In the introduction we posed a series of questions about the tempo of antigenic change in HIV within individuals and at the population level. Here we summarise our answers to those questions.

On average, how fast do HIV escape mutations arise in HLA matched individuals? We find that the median time to escape in HLA matched individuals across the 26 epitopes considered here is 8 years with an interquartile range of 1.8–34.0 years.

How fast do reversions occur in HLA mismatched people? Our inferred reversion rates are slow: there is no evidence of reversion in 56% of epitopes and at the lower quartile, the average time to reversion is 6.5 years.

These estimates, inferred using the model presented here from cross sectional population data, are highly consistent with independent estimates from an independent longitudinal cohort study. Taken together, these estimated rates of within-host evolution can accurately predict population level changes in the prevalence of escape mutants over the past 20 years for these 26 epitopes. The only data inconsistent with these estimates are the case studies which have driven the accumulated perception that escape is rapid and common. But case studies are subject to publication bias in favour of dramatic events and are not a reliable source of information on average rates of evolution across the population.

Is HIV still adapting to humans and if so how fast? We believe that HIV is still adapting to humans, and that the tempo of adaption will be different for different epitopes. We expect the prevalence of escape in some epitopes to carry on increasing far into the future, but only slowly.

What is the relationship between the tempo of adaptation within individuals and the rate of antigenic change across the entire pandemic? If HIV is still adapting, what patterns can we expect to unfold across the population of infected people? Depending on the rates of evolution within hosts we expect three different patterns of change in the prevalence of escape mutations. If the rate of reversion is zero then we expect the prevalence of escape mutations to carry on climbing, slowly, until they become fixed. If escape rates are fast or HLA prevalence is high then the rate of increase will be faster, but the expected qualitative pattern of slow rise in prevalence is independent of the escape rate. This pattern is illustrated in the curves in Figures 1C and 1D in which escape mutants never revert. It is perhaps counter-intuitive that it is epitopes with the very slowest reversion rates that we expect to display the most obvious population-level increases. For epitopes in which reversion is fast, or reversion and escape are both slow we expect the current prevalence of escape to be close to its long term level. Finally, if escape is fast and reversion is slow we expect the population prevalence of escape to fall as the underlying epidemic approaches its own long-term equilibrium. This third pattern is illustrated in the curve in Figure 1C with mean time to escape of 1 year and mean time to reversion of 10 years. These patterns are illustrated for particular parameter values in figure 1C and described for all possible parameter values in the analytic results presented in equations (S9) and (S12) of the Text S1.

How will these patterns be different in people of different HLA types in populations with different HLA frequencies? As one would intuitively expect, other things being equal, escape prevalence is lower in HLA mismatched people than HLA matched. It is also intuitively appealing that if the rate of reversion for an epitope is faster, then the discrepancy between prevalence in the two groups is greater. We also expect different dynamics in the HLA matched versus mismatched populations with escape prevalence in the two groups diverging in the years following the peak in the underlying epidemic. Of course this pattern is not what we expect if the reversion rate is zero in which case prevalence of escape slowly converges to fixation in both groups.

Finally, how will these patterns be different in populations with different HLA frequencies? The equations describing the temporary and final plateau in HLA matched, mismatched and total populations (Text S1, equations (S7)–(S12)) illustrate the intuitively appealing result that, other things being equal, escape prevalence increases with the underlying prevalence of HLA that restricts the escaping epitope.

Did our strict definition of escape bias our results towards those epitopes that evolve more slowly within hosts? We have explored the sensitivity of these results to different definitions of escaped epitopes and find that, if anything, our definition leads us to focus on epitopes that escape and revert more quickly. Nevertheless, this analysis only considers epitopes in gag, RT and nef restricted by HLA class I A and B alleles. HLA C-restricted epitopes [42] and epitopes in env and the accessory/regulatory genes may behave differently. Recent studies of the very first weeks of infection have described very early and rapid CTL escape in env in a small number of individuals [5], [43]. If these individuals are representative of the population, this intense early escape will be reflected in population prevalence of these env mutations. However the analysis presented here is a worked example of how events in a small number of individuals are not always representative of the wider population. Finally, as noted earlier, our escape rates are averaged across all HLA matched hosts. If only one third of HLA matched hosts actually mount a given epitope-specific response [33] our estimates of escape rates *in hosts who mount a response* would increase three-fold. This is not enough to reconcile the 20-fold difference between rates estimated from case-study data versus population data.

Model assumptions are another potential source of bias. We have presented a series of five additional models to check for the structural sensitivity of our findings. We find that none of the five different, more complex models we use to reinterpret the cross sectional data substantially alter our estimates of escape and reversion rates. These results are robust under several alternative models and different data definitions.

However we have not exhausted the infinite range of potential models we could use to better understand these questions. In future work we would hope to explore a number of further complications. Perhaps the most intriguing is to investigate the role of epistatic interactions between epitopes [44]. Although we have already considered overlapping epitopes, the role of more subtle, perhaps long-range interactions is clearly of great interest. We can also relax more of the assumptions of the simple model: that virulence and infectiousness are the same regardless of the host-virus pairing; that hosts can only ever transmit the type of virus which dominates their own infection; or that populations mix heterogeneously both socially and spatially. However, finding enough data to keep such complex models grounded in reality may be challenging.

This model provides a new framework with which to investigate how within-host evolution of CTL escape mutants translates to evolution of HIV at the population level. We have used it here to explain why some escape mutants have stable prevalence, whereas others continue to spread through the population. We have also used it to estimate within-host escape and reversion rates from population-level, cross-sectional sequence data. This is a useful tool since cross-sectional studies are faster, cheaper and often larger than longitudinal studies. Interpreting longitudinal studies is made difficult because mutants present at the first sample may have been transmitted or may have escaped prior to the first sample. Both cross-sectional data and this model include both means by which an individual can acquire an escape mutant and our rate estimates therefore account for them. In broader terms, this model allows comparisons across diverse sources of data on CTL escape. Although the model makes several simplifying assumptions, it reveals striking agreement across diverse and independent datasets: for most of the epitopes surveyed here, averaged across HLA matched individuals, escape happens slowly.

## Materials and Methods

### Ethics statement

This study has been approved by the Multicentre Research Ethics Committee (MREC). All patients provided written informed consent before participating in this study.

### Variables and parameters of the model

- *t* = time
- *h* = host type (0 if HLA mismatched, 1 if HLA matched)
- *v* = virus type (0 if wild-type (WT), 1 if escape mutant (EM))
- *B* = population birth rate (years^−1^)
- *p* = proportion of the population who are HLA matched

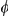
 = rate of escape in HLA matched hosts (years^−1^)
- *ψ* = rate of reversion in HLA mismatched hosts (years^−1^)
- *μ* = death rate of susceptible hosts (years^−1^)
- *α* = disease-related death rate (years^−1^)
- *β* = transmission probability per partnership
- *c* = rate of partner exchange (years^−1^)
- *X^h^(t)* = number of susceptible hosts of host type *h* at tim*e t*

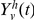
 = number of type *h* hosts infected with virus type *v* at time *t*
- *N(t)* = total number of hosts in the population at time *t*
- *λ_v_(t)* = force of infection from hosts infected with virus type *v* at time *t* (years^−1^)

### Model equations

The model is described mathematically using ordinary differential equations (1–6), where the force of infection is defined as 
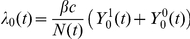
 for the wild-type virus and 
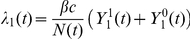
 for the escape mutant virus. The total population size is defined as 
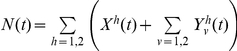
.

Susceptible, HLA mismatched
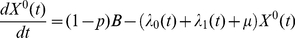
(1)


Susceptible, HLA matched
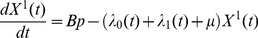
(2)


Infected, WT, HLA mismatched
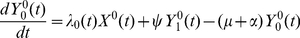
(3)


Infected, EM, HLA mismatched
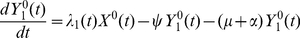
(4)


Infected, WT, HLA matched
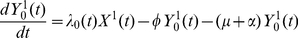
(5)


Infected, EM, HLA matched
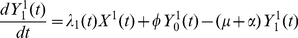
(6)


### Statistics

Minitab 14 was used to compare the observed and predicted escape rates (Figure 3A). Firstly, we tested whether they are correlated using a 2-tailed Pearson Correlation test. For this test, a small p-value (i.e. p-value<0.005) indicates a strong correlation. Since variables that are correlated are not necessarily equal in magnitude, we also evaluated whether the observed and predicted escape rates are approximately equal in magnitude. We present the t-value relating to a 2-tailed paired t-test with a null hypothesis that the difference in magnitude between the rates is equal to zero. For this test, a t-value with a small magnitude (i.e. less than 1) indicates that the variables are close in magnitude. Variables are typically regarded as statistically different in magnitude if the magnitude of the t-value is greater than 1.96. To meet the normality conditions for the two tests described, both sets of escape rates were first transformed according to 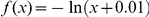. The observed and predicted changes in the escape prevalences (Figure 3C) were compared using the same tests, but each were first transformed according to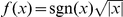. The observed and predicted reversion rates (Figure 3B) could not be normalised, therefore the correlation between these two variables was tested using the non-parametric Kendall Tau test. However, transformation of each set of reversion rates according to 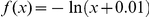 was sufficient to meet the normality conditions for the paired t-test.

## Supporting Information

Table S1 A summary of mutations in HIV-1 gag, RT and nef which have been reported in the literature as conferring CTL escape and confirmed by in vitro tests.
(0.02 MB PDF)

Table S2 A summary of escape and reversion data from the cross-sectional study (dataset 2) and the longitudinal cohort study (dataset 4).
(0.03 MB PDF)

Table S3 A summary of published escape data from case reports (dataset 3).
(0.03 MB PDF)

Table S4 A summary of published reversion data from case reports (dataset 3).
(0.01 MB PDF)

Text S1 Analytic expressions representing the escape prevalence under different circumstances.
(0.03 MB PDF)

Text S2 Supporting references.
(0.01 MB PDF)

Text S3 A detailed analysis describing how additional factors could affect the evolution of escape mutants and thus affect our inferred rates of escape and reversion rates from the cross-sectional data (dataset 2).
(0.41 MB PDF)

Figure S1 Confidence intervals for escape and reversion rates inferred from the cross-sectional data (dataset 2).
(0.04 MB PDF)

Figure S2 Sensitivity analysis showing how the assumed model parameters affect our inferences from the cross-sectional data (c.f. Figure 3).
(0.19 MB PDF)

Figure S3 Observed changes in the population escape prevalence over approximately 20 years (dataset 1).
(0.04 MB PDF)
