# Supplementary material for: Modelling the Evolution and Spread of HIV Immune Escape Mutants
Source: PLoS Pathog. 2010 Nov 18;6(11):e1001196. doi: 10.1371/journal.ppat.1001196 (PMC2987822; doi:10.1371/journal.ppat.1001196)
Supplement: Table S1 — A summary of mutations in HIV-1 gag, RT and nef which have been reported in the literature as conferring CTL escape and confirmed by in vitro tests. (0.02 MB PDF) [file ppat.1001196.s001.pdf]

HXB2 amino acid sequence	Author wildtype sequence if different to HXB2<br><br>B-clade ancestral sequence if different to HXB2	Gene (HXB2 [S1])	Amino acid location (HXB2 [S1])	HLA	Proportion of Caucasians with HLA [36] (p)	Escape mutations[b]	Reference
KIRLRPGGK	-	p17 gag	18-26	A3	0.224	K9R<br>K1R & K9R	Wilson 1999 [S2], Sanchez-Merino 2005 [S3]<br>Wilson 1999 [S2]
RLRPGGKKK	-	p17 gag	20-28	A3	0.224	K9Q<br>K9T<br>K9R	Allen 2004 [6]<br>Allen 2004 [6]<br>Geels 2003 [19]
GGKKKYKL	-	p17 gag	24-32	B8	0.143	K3R<br>K5Q<br>K7R<br>K7Q<br>K5Q & K7R<br>K3R & K5T	Reid 1996 [S4]<br>Price 1997 [3]<br>Reid 1996 [S4]<br>Reid 1996 [S4]<br>Milicic 2005 [S5]<br>Milicic 2005 [S5]
KYKLKHIVW	-	p17 gag	28-36	A24	0.196	K1Q<br>K3R & I7L<br>K1R & K3R & I7L	Yokomaku 2004 [S6]<br>Yokomaku 2004 [S6]<br>Yokomaku 2004 [S6]
GSEELRSLY	-	p17 gag	71-79	A1	0.262	L5I & R6K<br>Y9F	Milicic 2005 [S5]<br>Milicic 2005 [S5]
SLYNTVATL	-	p17 gag	77-85	A2	0.438	Y3F<br>N4I<br>T5A<br>T5L<br>V6I<br>T8V<br>Y3H & T8V	Jamieson 2003 [S7]<br>Brander 1998 [S8]<br>Brander 1999 [S9]<br>Brander 1998 [S8]<br>Geels 2006 [S10]<br>Jamieson 2003 [S7]<br>Jamieson 2003 [S7]
AADTGHSNQ[a]	AADTG**N**S**S**Q<br>AADTG**N**S**S**Q	p17 gag	119-127	Not determined		many<br>(see paper)	Jones 2004 [S11]
HSNQVSQNYP[a]	**N**S**S**QVSQNYP<br>**N**S**S**QVSQNYP	p17 gag	124-133	Not determined		many<br>(see paper)	Jones 2004 [S11]
ISPRTLNAW	-	p24 gag	15-23	B57	0.057	A(-1)P<br>I1M<br>I1L	Draenert 2004 [S12]<br>Pillay 2005 [S13]<br>Pillay 2005 [S13]
KAFSPEVIPMF	-	p24 gag	30-40	B57	0.057	A2G	Crawford 2007 [S14]
EVIPMFSAL	-	p24 gag	35-43	A26	0.083	E1K<br>S7A<br>S7T	Kawashima 2005 [S15]<br>Kawashima 2005 [S15]<br>Kawashima 2005 [S15]
TSTLQEQIGW	HXB2 and<br>TSTLQEQI**A**W	p24 gag	108-117	B57/B58	0.096	T3N	Leslie 2004 [17], Allen 2004 [6], Feeney 2005 [S16], Pillay 2005 [S13]
KRWIILGLNK	-	p24 gag	131-140	B27	0.073	R2K<br>R2G<br>R2Q	Kelleher 2001 [20], Goulder 1997 [8], Geels 2006 [S10]<br>Kelleher 2001 [20]<br>Ammarnond 2005 [S17]
NANPDCKTI	-	p24 gag	193-201	B51	0.126	A2S & T8N	Yokomaku 2004 [S6]
DCKTILKAL	-	p24 gag	197-205	B8	0.143	K3R	Nowak 1995 [21]
CGKEGHTAR	CGKEGH**I**AR<br>CGKEGH**I**AR	p7 gag	32-40	A31	0.050	R9K	Goonetilleke 2009 [5]
GPKVKQWPL	-	RT	18-26	B8	0.143	K3R<br>K3E	Meier 1995 [S18]<br>Meier 1995 [S18]
TAFTIPSI	-	RT	128-135	B51	0.126	I8T	Geels 2006 [S10]
AIFQSSMTK	-	RT	158-166	A3/A11	0.340	Q4L<br>A1T & F3S	Wilson 1999 [S2]<br>Wilson 1999 [S2]
IVLPEKDSW	-	RT	244-252	B57	0.057	V2M<br>V2T<br>D7G	Menendez-Arias 1998 [S19]<br>Menendez-Arias 1998 [S19]<br>Menendez-Arias 1998 [S19]
IPLTEEAEL[a]	**V**PLTEEAEL	RT	293-301	B57	0.057	E5V<br>E5A<br>E5G	Oxenius 2004 [S20]
ETFYVDGAANR[a]	-	RT	438-448	A26	0.083	F3Y & D6N	Wilson 1999 [S2]
QVPLRPMTYK	-	nef	73-82	A3/A11	0.340	V2A<br>L4A<br>R5A<br>M7A<br>T8A	Milicic 2005 [S5]<br>Keonig 1995 [S21]<br>Koenig 1995 [S21]<br>Koenig 1995 [S21]<br>Koenig 1995 [S21]
VPLRPMTY	-	nef	74-81	B35	0.196	T7S<br>Y8F	Oxenius 2004 [S20]<br>Oxenius 2004 [S20]
RPMTYKAAV	RPMTYK**G**A**L**<br>RPMTYKAA**L**	nef	77-85	B7	0.166	T4S<br>Y5F	Oxenius 2004 [S20]<br>Oxenius 2004 [S20]
KAAVDLSHF	KAA**L**DLSHF	nef	82-90	B57/B58	0.096	A2G	Leslie 2005 [S22]
FLKEKGGL	-	nef	90-97	B8	0.143	E4D<br>K5N<br>K5E<br>L2I & K5N<br>K3E & K5N<br>E4G & K5N	Price 1997 [3]<br>Price 1997 [3]<br>Price 1997 [3]<br>Price 1997 [3]<br>Price 1997 [3]<br>Price 1997 [3]
HTQGYFPDW	-	nef	116-125	B57	0.057	H1N	Pillay 2005 [S13]
TPGPGVRYPL	TPGPG**I**RYPL	nef	128-137	B42/B7	0.169	P2S<br>P2Q	Pillay 2005 [S13]<br>Pillay 2005 [S13]
RYPLTFGWCY	RYPLTFGWC**F**<br>RYPLTFGWC**F**	nef	134-143	A24	0.196	Y2F	Furutsuki 2004 [S23]
SRLAFHHVAR[a]	SRLAFHH**M**AR<br>S**S**LAFHHVAR	nef	187-196	A31	0.050	R10Q	Goonetilleke 2009 [5]

Table S1. A summary of mutations in HIV-1 gag, RT and nef which have been reported in the literature as conferring CTL escape and confirmed by *in vitro* tests.

In some cases the HBX2 epitope sequence differs from the author wildtype sequence and/or the B-clade ancestral sequence [S24]. These changes are shown in column 2. Note that we have not included in this list any defined escape mutations that lie in sites that differ between the author wildtype sequence and the B-clade ancestral sequence. Epitopes are ordered according to their location within the genome. The proportion of Caucasians with each HLA type is estimated from allele frequencies amongst Caucasians [36], assuming mendelian genetics. This calculation ignores linkage between alleles. [a]Epitopes AADTGHSNQ and HSNQVSQNYP are excluded from analysis of the cross-sectional data (dataset 2) and the longitudinal cohort data (dataset 4) because their HLA-restrictions were not determined. In addition, sequence data were not available from the cross-sectional study for IPLTEEAEL or SRLAFHHVAR or from the longitudinal cohort study for ETFYVDGAANR. Sequence data for CGKEGHTAR were available from neither study. [b]Where two or more different mutations are given together with the '&' sign, it indicates that an epitope containing that combination of mutations was tested and found to confer escape.
