# Supplementary material for: Modelling the Evolution and Spread of HIV Immune Escape Mutants
Source: PLoS Pathog. 2010 Nov 18;6(11):e1001196. doi: 10.1371/journal.ppat.1001196 (PMC2987822; doi:10.1371/journal.ppat.1001196)
Supplement: Table S2 — A summary of escape and reversion data from the cross-sectional study (dataset 2) and the longitudinal cohort study (dataset 4). (0.03 MB PDF) [file ppat.1001196.s002.pdf]

HXB2 Amino Acid sequence	Escape site	Cross-sectional study (dataset 2)				Longitudinal cohort study (dataset 4)			
		HLA matched with escape	HLA mismatched with escape	Estimated time to escape (years)	Estimated time to reversion (years)	HLA matched with escape	HLA mismatched with escape	Estimated time to escape (years)	Estimated time to reversion (years)
KIRLRPGGK	K1, K9	4/14	7/32	23.9	No rev	3/14	2/38	10.5	36.4
RLRPGGKKK	K9	8/14	5/32	4.42	5.66	5/10	7/45	2.15	13.0
GGKKKYKL	K3, K5, K7	6/7	28/39	1.64	No rev	2/7	4/101	4.13	>50
KYKLKHIVW	K1, K3, I7	8/9	26/37	1.76	No rev	5/5	4/106	0.75	>50
GSEELRSLY	L5, R6, Y9	10/11	24/35	1.73	>50	4/7	2/91	2.38	>50
SLYNTVATL	Y3, N4, T5, V6, T8	8/14	19/32	14.7	No rev	2/25	3/55	27.8	34.2
AADTGHSNQ	A1, G5, N6, S7, S8, Q9	HLA restriction not defined							
HSNQVSQNYP	N1, S2, S3, Q4, V5	HLA restriction not defined							
ISPRTLNAW	A(-1), I1	6/8	21/39	1.75	No rev	3/5	4/75	1.92	37.7
KAFSPEVIPMF	A2	0/10	5/66	>50	No rev	1/9	0/6	16.0	No rev
EVIPMFSAL	E1, S7	1/4	16/72	16.6	No rev	1/4	0/43	6.75	No rev
TSTLQEQIGW	T3	10/15	4/61	2.04	3.78	4/6	7/12	1.56	2.50
KRWIILGLNK	R2	3/9	3/67	8.28	7.37	3/17	1/9	11.1	14.5
NANPDCKTI	A2, T8	2/11	13/65	29.6	No rev	0/9	2/30	No esc	29.8
DCKTILKAL	K3	2/14	1/62	26.3	3.00	0/35	0/1	No esc	No rev
CGKEGHTAR	R9	No sequence				No sequence			
GPKVKQWPL	K3	1/8	4/45	48.5	No rev	0/12	0/8	No esc	No rev
TAFTIPSI	I8	7/8	20/55	0.93	25.2	1/1	2/28	0.50	24.6
AIFQSSMTK	A1, F3, Q4	2/22	1/41	>50	3.66	0/22	0/2	No esc	No rev
IVLPEKDSW	V2, D7	4/6	19/55	2.58	No rev	1/1	0/30	0.50	No rev
IPLTEEAEL	E5	No sequence				0/1	1/21	No esc	37.5
ETFYVDGAANR	F3, D6	0/3	0/71	No esc	Indeterminate	No sequence			
QVPLRPMTYK	V2, L4, R5, M7, T8	0/13	2/38	>50	No rev	2/21	5/11	18.4	1.85
VPLRPMTY	T7, Y8	3/7	2/44	6.18	2.02	3/9	4/18	4.50	6.00
RPMTYKAAV	T4, Y5	1/5	4/46	26.2	25.2	2/18	2/16	16.4	12.4
KAAVDLSHF	A2	4/8	21/43	6.25	No rev	1/2	7/58	3.50	14.0
FLKEKGGL	L2, K3, E4, K5	4/8	12/43	7.83	No rev	-/0	0/88	Indeterminate	No rev
HTQGYFPDW	H1	6/6	11/45	0.00	27.7	2/5	0/32	2.25	No rev
TPGPGVRYPL	P2	0/5	1/46	>50	No rev	1/22	1/5	34.5	9.00
RYPLTFGWCY	Y2	12/14	7/37	0.89	4.83	5/11	5/18	1.85	6.65
SRLAFHHVAR	R10	No sequence				1/3	4/45	3.50	14.7

Table S2. A summary of escape and reversion data from the cross-sectional study (dataset 2) and the longitudinal cohort study (dataset 4).

This is a summary of escape and reversion data relating to 31 epitopes with described escape mutants in HIV-1 gag, RT and nef. Sequence data from other HIV genes were not yet available from either of these studies. These are the same epitopes (in the same order) as provided in Table S1. In this analysis any mutation in any of the previously defined escape sites within an epitope (Table S1) was counted as an escape mutant. The prevalence of escape in HLA matched and HLA mismatched hosts in the cross-sectional data are provided here in columns 3 and 4 and also in Fig. 2B. Escape and reversion rates estimated from these data using the mathematical model are presented in columns 5-8. Model estimates assume that $\beta c = 0.3$ (i.e. $R_0 = 3$ if $\mu + \alpha = 0.1$), that the epidemic duration is 27 years (t=27) and that the proportion of hosts in the population with each HLA is as presented in Table S1. The escape rate estimates in columns 5 and 9 are compared in Fig. 3A. Likewise, the reversion rate estimates in columns 6 and 10 are compared in Fig. 3B.
