# Supplementary material for: Modelling the Evolution and Spread of HIV Immune Escape Mutants
Source: PLoS Pathog. 2010 Nov 18;6(11):e1001196. doi: 10.1371/journal.ppat.1001196 (PMC2987822; doi:10.1371/journal.ppat.1001196)
Supplement: Table S3 — A summary of published escape data from case reports (dataset 3). (0.03 MB PDF) [file ppat.1001196.s003.pdf]

HXB2 amino-acid sequence	Author wildtype sequence if different to HXB2	Gene (HXB2 [S1])	Amino acid location (HXB2 [S1])	HLA restriction	Escape mutations (away from author wildtype)	Time between infection and escape[a] (years)	Time between infection and last sample without escape (years)	Estimated average time to escape[b] (years^{-1})	Number of patients	Number of patients with escape	Reference {number of patients per reference}
RLRPGGKKK	-	p17 gag	20-28	A3	K9R, K9Q, K9T	1.15, 1.71[c], 2.15, 2.39[c]		1.15	4	4	Allen 2004 [6]{3}, Geels 2003 [19] {1}
SLYNTVATL	-	p17 gag	77-85	A2	T8V, V6I	3.53, 4.38[c], 7.31, 8.50[c]		3.53	4	4	Jamieson 2003 [S7] {3}, Geels 2003 [19] {1}
AADTGHSNQ	AADTG**N**S**S**Q	p17 gag	119-127	Not determined	several (see paper)	0.25		0.25	1	1	Jones 2004 [S11]
HSNQVSQNYP	**N**S**S**QVSQNYP	p17 gag	124-133	Not determined	several (see paper)	0.39		0.39	1	1	Jones 2004 [S11]
ISPRTLNAW		p24 gag	15-23	B57	I1M, I1L	0.28, 0.41	0.42	0.41	3	2	Pillay 2005 [S13] {1}, Goonetilleke 2009 [5] {2}
TSTLQEQIGW	TSTLQEQIGW TSTLQEQI**A**W	p24 gag	108-117	B57/B58	T3N	0.17, 0.35, 0.99, 1.00, 1.02, 1.60, 2.00[c], 2.50, 3.00[c], 5.50[c]	6.00	1.00	11	10	Leslie 2004 [6] {6}, Feeney 2005 [S16] {2}, Allen 2004 [6] {1}, Pillay 2005 [S13] {1}, Goonetilleke 2009 [5] {1}
KRWIILGLNK	-	p24 gag	131-140	B27	R2K, R2G	0.85, 3.78[c], 7.30, 7.96, 12.42	0.90, 1.18, 1.28, 7.33, 9.90, 14.10, 14.50, 15.40	12.42	13	5	Goulder 1997 [8] & Kelleher 2001 [20] {5}, Kelleher 2001 [20] {6}, Goulder 1997 [8] {1}, Geels 2006 [S10] {1}
CGKEGHTAR	CGKEGH**I**AR	p7 gag	32-40	A31	I7L, R9K	0.12		0.12	1	1	Goonetilleke 2009 [5]
TAFTIPSI	-	RT	128-135	B51	I8T	2.67		2.67	1	1	Geels 2003 [19]
IPLTEEAEL	-	RT	293-301	B57	E5V, E5A E5G	1.00		1.00	1	1	Oxenius 2004 [S20]
VPLRPMTY	-	nef	74-81	B35	T7S, Y8F	1.50		1.50	1	1	Oxenius 2004 [S20]
RPMTYKAAV	RPMTYK**G**A**L**	nef	77-85	B7	T4S, Y5F	1.19		1.19	1	1	Oxenius 2004 [S20]
KAAVDLSHF	-	nef	82-90	B57/B58	A2G	0.28, 0.32, 0.68, 3.00[c], 2.00[c]		0.32	5	5	Leslie 2005 [S22] {3}, Pillay 2005 [S13] {1}, Goonetilleke 2009 [5] {1}
FLKEKGGL	-	nef	90-97	B8	K5N, K5E, E4D, (L2I &K5N), (K3E & K5N), (K4G & K5N)	0.24		0.24	1	1	Price 1997 [3]
HTQGYFPDW	-	nef	116-124	B57	H1N	0.35	1.04	0.35	2	1	Pillay 2005 [S13] {1}, Goonetilleke 2009 [5] {1}
TPGPGVRYPL	-	nef	128-137	B42	P2S, P2Q	0.43		0.43	1	1	Pillay 2005 [S13]
RYPLTFGWCY	RYPLTFGWC**F**	nef	134-143	A24	Y2F	0.25		0.25	1	1	Sanchez-Merino 2005 [S3]
SRLAFHHVAR	S**S**LAFHHVAR	nef	187-196	A31	S2N, R10Q	0.02		0.02	1	1	Goonetilleke 2009 [5]

HXB2 amino-acid sequence	Author amino-acid sequence if different to HXB2	HXB2 Gene	Amino acid location (HXB2)	HLA restriction	Escape mutation (away from author wildtype)	Time between infection and escape[a] (years)	Time between infection and last sample without escape (years)	Estimated average time to escape[b] (years)	Number of patients	Number of patients with escape	Reference (number of patients per reference)
TEKLWVTVY	**A**E**N**LWVTVY	env gp160	31-39	B44	several (see paper)	0.15		0.15	1	1	Jones 2004 [S11] & Borrow 1997 [2]
DPNPQEVVL	DPNPQEV**A**L	env gp160	78-86	B35	A8P, V7A	0.76		0.76	1	1	Oxenius 2004 [S20]
SFEPIPIHY	-	env gp160	209-217	A29	S1T, E4D, (F2I & Y9F)	0.33	2.19	0.33	2	1	Jones 2004 [S11]
QFGNNKTIIF	QF**R**N_**Y**TIVF	env gp160	352-361	Cw4	R3K	0.04		0.04	1	1	Goonetilleke 2009 [5]
SFNCGGEFF	-	env gp160	375-383	B15	G5R	0.45		0.45	1	1	Geels 2003 [19]
ERYLKDQQL	-	env gp160	584-592	B14	K5T, K5Q, K5S, K5M, Y3H	0.44, 0.48, 1.16		0.69	3	3	Jones 2004 [S11] {2}, Goonetilleke 2009 [5] {1}
EAVRHFPRI	-	vpr	29-37	B51	I9T<br>I9L	0.42		0.42	1	1	Cao 2003 [S25]
CCFHCQVC	-	tat	30-37	Cw12	F3L<br>V7S	0.20	0.91	0.57	2	1	Cao 2003 [S25]
NCYCKKCCF	NCYCKKCCF<br>NCYCKKCC**Y**	tat	24-32	A29	N1K<br>N1K & K6R<br>N1K & K6Q	0.16, 0.23		0.19	2	2	Jones 2004 [S11]
FHCQVCFITK	FHCQVCF**M**TK	tat	32-41	Not determined	F1L & T9K	0.23		0.23	1	1	Jones 2004 [S11]

Table S3. A summary of published escape data from case reports (dataset 3).

A summary of published data relating the appearance of escape mutants, as presented in Fig. 2C (dataset 3). Mutations are included in this summary if they have been described as conferring escape and confirmed *in vitro* somewhere in the published literature. Patient-epitope pairs are included if three conditions are met: 1) an estimate is provided for the infection date or time of seroconversion of the patient, 2) if the patient is HLA matched for the epitope in question and 3) if the patient has 0% escape mutant at the first sample. This table includes all of the 18 epitopes in Table S1 for which case report data of this specification are available. Note that unlike Table S1 this dataset is not restricted to epitopes in gag, RT and Nef. It includes data from an additional 10 epitopes with known escape mutations in env, vpr and tat. Epitopes are ordered according to their location within the genome. [a]For patient-epitope pairs for which escape mutants arose during the study period, the time between infection and escape is estimated using linear interpolation to be the first time-point at which 50% of the patient's sequences were mutant. Patients who were tracked from the time that they seroconverted were assumed to have become infected 30 days prior to seroconversion. [b]For each epitope the average time between infection and escape is estimated by summing across patients all the times to escape (column 7) and the times to the last sample without escape (column 8) and dividing by the number of patients in whom escape mutants occurred. [c]For patients in whom escape mutants occurred, if the time between the last sample without escape and the first sample with escape was more than 2 years the data were excluded from the analysis of the average time to escape.
