# Supplementary material for: Modelling the Evolution and Spread of HIV Immune Escape Mutants
Source: PLoS Pathog. 2010 Nov 18;6(11):e1001196. doi: 10.1371/journal.ppat.1001196 (PMC2987822; doi:10.1371/journal.ppat.1001196)
Supplement: Table S4 — A summary of published reversion data from case reports (dataset 3). (0.01 MB PDF) [file ppat.1001196.s004.pdf]

| Amino-acid sequence (HXB2) | Author amino-acid sequence if different to HXB2 | Gene (HXB2) | Amino acid location (HXB2) | HLA restriction | Escape mutation | Time between infection and reversion[a] | Time between infection and last sample without reversion | Estimated time to reversion[b] | Number of patients | Number of patients with reversion | Author |
|---|---|---|---|---|---|---|---|---|---|---|---|
| KIRLRPGGK<br>GGKKKYKL | | p17 gag | 18-26<br>24-31 | A3<br>B8 | K9R<br>K3R | 0.78 | 1.00 | 0.78 | 2 | 1 | Sanchez-Merino 2005 [S3] {1}, Milicic 2005 [S5] {1} |
| ISPRTLNAW | | p24 gag | 15-23 | B57 | A(-1)P | | 8.00 | >8.00 | 1 | 0 | Draenert 2004 [S12] |
| TSTLQEQIGW | | p24 gag | 108-117 | B57/5801 | T3N | 0.58, 1.67 | | 0.58 | 2 | 2 | Leslie 2004 [17] |
| KAAVDLSHF | KAAFDLSFF | nef | 82-90 | B57/5801 | A2G | | 2.00, 2.00 | >2.00 | 2 | 0 | Leslie 2005 [S22] |
| LPPVVAKEI | | integrase | 28-36 | B51 | V4I | | 0.66, 1.15 | >1.15 | 2 | 0 | Leslie 2005 [S22] |

## Table S4. A summary of published reversion data from case reports (dataset 3).

A summary of published data relating the reversion of escape mutants, as presented in Fig. 2D (dataset 3). Mutations are included in this summary if they have been described as conferring escape and confirmed *in vitro* in the literature. Patient-epitope pairs are included if three conditions are met: 1) an estimate is provided for the infection date or time of seroconversion of the patient, 2) if the patient is HLA mismatched for the epitope in question and 3) if the patient has 100% escape mutant at the first sample. [a]For patient-epitope pairs for which escape mutants reverted during the study period, the time between infection and reversion is estimated using linear interpolation to be the first time-point at which 50% of the patient's sequences were mutant. Patients who were tracked from the time that they seroconverted were assumed to have become infected 30 days prior to seroconversion. [b]For each epitope the average time between infection and reversion is estimated by summing across patients all the times to reversion (column 7) and the times to the last sample without reversion (column 8) and dividing by the number of patients in whom reversion occurred.
