# Supplementary material for: Modelling the Evolution and Spread of HIV Immune Escape Mutants
Source: PLoS Pathog. 2010 Nov 18;6(11):e1001196. doi: 10.1371/journal.ppat.1001196 (PMC2987822; doi:10.1371/journal.ppat.1001196)
Supplement: Text S1 — Analytic expressions representing the escape prevalence under different circumstances. (0.03 MB PDF) [file ppat.1001196.s005.pdf]

Text S1. Analytic expressions representing the escape prevalence under different circumstances.

Expression are provided for the escape prevalence in HLA matched hosts, $Y_1^1 \big/ (Y_0^1 + Y_1^1)$; HLA mismatched hosts, $Y_1^0 \big/ (Y_0^0 + Y_1^0)$; and all hosts $(Y_1^0 + Y_1^1) \big/ (Y_1^0 + Y_1^1 + Y_0^0 + Y_0^1)$.

<u>During the exponential phase of the epidemic</u>

HLA matched hosts

$$\frac{\phi}{\psi\beta c(1-p)+\phi\psi+\phi\beta cp}\left((\psi+\beta cp) - \frac{e^{-\frac{1}{2}(\beta c+\phi+\psi)t}}{\varpi}\Big(\big(\beta^2 c^2 p(1-p) + \bar{\varepsilon}(\psi+\beta cp)\big) e^{\frac{1}{2}\varpi t} - \big(\beta^2 c^2 p(1-p) + \varepsilon(\psi+\beta cp)\big) e^{-\frac{1}{2}\varpi t} \Big) \right) \quad \text{(S1)}$$

HLA mismatched hosts

$$\frac{\phi}{\psi\beta c(1-p)+\phi\psi+\phi\beta cp}\left(\beta cp + \frac{e^{-\frac{1}{2}(\beta c+\phi+\psi)t}}{\beta c(1-p)\varpi}\Big(\varepsilon\big(\beta^2 c^2 p(1-p) + \bar{\varepsilon}(\psi+\beta cp)\big) e^{\frac{1}{2}\varpi t} - \bar{\varepsilon}\big(\beta^2 c^2 p(1-p) + \varepsilon(\psi+\beta cp)\big) e^{-\frac{1}{2}\varpi t} \Big) \right) \quad \text{(S2)}$$

All hosts

$$\frac{\phi}{\psi\beta c(1-p)+\phi\psi+\phi\beta cp}\left(p(\psi+\beta c) + \frac{e^{-\frac{1}{2}(\beta c+\phi+\psi)t}}{\beta c\varpi}\Big((\varepsilon-\beta cp)\big(\beta^2 c^2 p(1-p) + \bar{\varepsilon}(\psi+\beta cp)\big) e^{\frac{1}{2}\varpi t} - (\bar{\varepsilon}-\beta cp)\big(\beta^2 c^2 p(1-p) + \varepsilon(\psi+\beta cp)\big) e^{-\frac{1}{2}\varpi t} \Big) \right) \quad \text{(S3)}$$

Where $\varpi = \sqrt{(\beta c - \phi + \psi)^2 + 4\beta c(1-p)(\phi-\psi)} > 0$ (S4)

$\varepsilon = \frac{1}{2}\big(\psi - \phi - \varpi - \beta c(1-2p)\big)$ (S5)

$\bar{\varepsilon} = \frac{1}{2}\big(\psi - \phi + \varpi - \beta c(1-2p)\big)$ (S6)

<u>At the temporary plateau</u>

HLA matched hosts $\dfrac{\phi(\psi+\beta cp)}{\psi\beta c(1-p)+\phi\psi+\phi\beta cp}$ (S7)

HLA mismatched hosts $\dfrac{\phi\beta cp}{\psi\beta c(1-p)+\phi\psi+\phi\beta cp}$ (S8)

All hosts $\dfrac{\phi p(\psi+\beta c)}{\psi\beta c(1-p)+\phi\psi+\phi\beta cp}$ (S9)

At the long term equilibrium

HLA matched hosts $$\frac{\phi\left(\psi+(\mu+\alpha)p\right)}{\psi(\mu+\alpha)(1-p)+\phi\psi+\phi(\mu+\alpha)p} \quad \text{(S10)}$$

HLA mismatched hosts $$\frac{\phi\left(\mu+\alpha\right)p}{\psi(\mu+\alpha)(1-p)+\phi\psi+\phi(\mu+\alpha)p} \quad \text{(S11)}$$

All hosts $$\frac{\phi p(\psi+\mu+\alpha)}{\psi(\mu+\alpha)(1-p)+\phi\psi+\phi(\mu+\alpha)p} \quad \text{(S12)}$$

Equations S1-S3 represent the escape prevalences during the exponential growth phase of the epidemic. They are derived by assuming that early on during an epidemic the total number of hosts in the population is equal to the number of susceptibles, i.e. $N(t)=X(t)$. This assumption simplifies the model (equations 1-6 of the main text) to a set of first order linear differential equations, which can be solved analytically. At the start of the epidemic it is assumed that no hosts are infected with the escape mutant and that a proportion, *p*, of both susceptible and infected hosts are HLA matched. Thus if $X(0)$ and $Y(0)$ represent the total number of susceptible and infected hosts at the start of the epidemic, the initial conditions were as follows: $X^1(0)=pX(0)$, $X^0(0)=(1-p)X(0)$, $Y_0^1(0)=pY(0)$, $Y_0^0(0)=(1-p)Y(0)$ and $Y_1^1(0)=Y_1^0(0)=0$. In addition, the population size is assumed to remain constant in the absence of infection, thus $B=\mu X(0)$.

Equations S7-S9 are the time-independent terms from the escape prevalences during the exponential growth phase of the epidemic (equations S1-S3). More formally, they can be derived from equations 1-3, as the limit as the epidemic duration (*t*) gets very large. These expressions approximate the escape prevalences during the latter stages of the exponential phase. It can be shown numerically that they are a better approximation when the reversion rate is faster in comparison to the escape rate. Under such circumstances the escape prevalences reach temporary plateaus. Thus expressions S7-S9 define the escape prevalences at the temporary plateaus.

Equations S10-S12 represent the escape prevalences at the long term equilibria and can be derived by setting $dX^h/dt=0$ and $dY_v^h/dt=0$ for each host and virus type.
