# Supplementary material for: Modelling the Evolution and Spread of HIV Immune Escape Mutants
Source: PLoS Pathog. 2010 Nov 18;6(11):e1001196. doi: 10.1371/journal.ppat.1001196 (PMC2987822; doi:10.1371/journal.ppat.1001196)
Supplement: Text S2 — Supporting references. (0.01 MB PDF) [file ppat.1001196.s006.pdf]

Text S2. Supporting references

S1. Korber BT, Foley BT, Kuiken CL, Pillai SK, Sodroski JG Numbering Positions in HIV Relative to HXB2CG. www.hiv.laln.gov/content/sequence/HIV/REVIEWS/HXB2.

S2. Wilson CC, Brown RC, Korber BT, Wilkes BM, Ruhl DJ, et al. (1999) Frequent detection of escape from cytotoxic T-lymphocyte recognition in perinatal human immunodeficiency virus (HIV) type 1 transmission: the ariel project for the prevention of transmission of HIV from mother to infant. J Virol 73: 3975-3985.

S3. Sanchez-Merino V, Nie S, Luzuriaga K (2005) HIV-1-specific CD8+ T cell responses and viral evolution in women and infants. J Immunol 175: 6976-6986.

S4. Reid SW, McAdam S, Smith KJ, Klenerman P, O'Callaghan CA, et al. (1996) Antagonist HIV-1 Gag peptides induce structural changes in HLA B8. J Exp Med 184: 2279-2286.

S5. Milicic A, Edwards CT, Hue S, Fox J, Brown H, et al. (2005) Sexual transmission of single human immunodeficiency virus type 1 virions encoding highly polymorphic multisite cytotoxic T-lymphocyte escape variants. J Virol 79: 13953-13962.

S6. Yokomaku Y, Miura H, Tomiyama H, Kawana-Tachikawa A, Takiguchi M, et al. (2004) Impaired processing and presentation of cytotoxic-T-lymphocyte (CTL) epitopes are major escape mechanisms from CTL immune pressure in human immunodeficiency virus type 1 infection. J Virol 78: 1324-1332.

S7. Jamieson BD, Yang OO, Hultin L, Hausner MA, Hultin P, et al. (2003) Epitope escape mutation and decay of human immunodeficiency virus type 1-specific CTL responses. J Immunol 171: 5372-5379.

S8. Brander C, Hartman KE, Trocha AK, Jones NG, Johnson RP, et al. (1998) Lack of strong immune selection pressure by the immunodominant, HLA-A*0201-restricted cytotoxic T lymphocyte response in chronic human immunodeficiency virus-1 infection. J Clin Invest 101: 2559-2566.

S9. Brander C, Yang OO, Jones NG, Lee Y, Goulder P, et al. (1999) Efficient processing of the immunodominant, HLA-A*0201-restricted human immunodeficiency virus type 1 cytotoxic T-lymphocyte epitope despite multiple variations in the epitope flanking sequences. J Virol 73: 10191-10198.

S10. Geels MJ, Jansen CA, Baan E, De Cuyper IM, van Schijndel GJ, et al. (2006) CTL escape and increased viremia irrespective of HIV-specific CD4+ T-helper responses in two HIV-infected individuals. Virology 345: 209-219.

S11. Jones NA, Wei X, Flower DR, Wong M, Michor F, et al. (2004) Determinants of human immunodeficiency virus type 1 escape from the primary CD8+ cytotoxic T lymphocyte response. J Exp Med 200: 1243-1256.

S12. Draenert R, Allen TM, Liu Y, Wrin T, Chappey C, et al. (2006) Constraints on HIV-1 evolution and immunodominance revealed in monozygotic adult twins infected with the same virus. J Exp Med 203: 529-539.

S13. Pillay T, Zhang HT, Drijfhout JW, Robinson N, Brown H, et al. (2005) Unique acquisition of cytotoxic T-lymphocyte escape mutants in infant human immunodeficiency virus type 1 infection. J Virol 79: 12100-12105.

S14. Crawford H, Prado JG, Leslie A, Hue S, Honeyborne I, et al. (2007) Compensatory mutation partially restores fitness and delays reversion of escape mutation within

the immunodominant HLA-B*5703-restricted Gag epitope in chronic human immunodeficiency virus type 1 infection. J Virol 81: 8346-8351.

S15. Kawashima Y, Satoh M, Oka S, Takiguchi M (2005) Identification and characterization of HIV-1 epitopes presented by HLA-A*2603: comparison between HIV-1 epitopes presented by A*2601 and A*2603. Hum Immunol 66: 1155-1166.

S16. Feeney ME, Tang Y, Pfafferott K, Roosevelt KA, Draenert R, et al. (2005) HIV-1 viral escape in infancy followed by emergence of a variant-specific CTL response. J Immunol 174: 7524-7530.

S17. Ammaranond P, Zaunders J, Satchell C, van Bockel D, Cooper DA, et al. (2005) A new variant cytotoxic T lymphocyte escape mutation in HLA-B27-positive individuals infected with HIV type 1. AIDS Res Hum Retroviruses 21: 395-397.

S18. Meier UC, Klenerman P, Griffin P, James W, Koppe B, et al. (1995) Cytotoxic T lymphocyte lysis inhibited by viable HIV mutants. Science 270: 1360-1362.

S19. Menendez-Arias L, Mas A, Domingo E (1998) Cytotoxic T-lymphocyte responses to HIV-1 reverse transcriptase (review). Viral Immunol 11: 167-181.

S20. Oxenius A, Price DA, Trkola A, Edwards C, Gostick E, et al. (2004) Loss of viral control in early HIV-1 infection is temporally associated with sequential escape from CD8+ T cell responses and decrease in HIV-1-specific CD4+ and CD8+ T cell frequencies. J Infect Dis 190: 713-721.

S21. Koenig S, Conley AJ, Brewah YA, Jones GM, Leath S, et al. (1995) Transfer of HIV-1-specific cytotoxic T lymphocytes to an AIDS patient leads to selection for mutant HIV variants and subsequent disease progression. Nat Med 1: 330-336.

S22. Leslie A, Kavanagh D, Honeyborne I, Pfafferott K, Edwards C, et al. (2005) Transmission and accumulation of CTL escape variants drive negative associations between HIV polymorphisms and HLA. J Exp Med 201: 891-902.

S23. Furutsuki T, Hosoya N, Kawana-Tachikawa A, Tomizawa M, Odawara T, et al. (2004) Frequent transmission of cytotoxic-T-lymphocyte escape mutants of human immunodeficiency virus type 1 in the highly HLA-A24-positive Japanese population. J Virol 78: 8437-8445.

S24. Cooper LJ, Wacker DP, McComas JJ, Brown K, Peck SM, et al. (1995) Use of component analyses to identify active variables in treatment packages for children with feeding disorders. J Appl Behav Anal 28: 139-153.

S25. Cao J, McNevin J, Malhotra U, McElrath MJ (2003) Evolution of CD8+ T cell immunity and viral escape following acute HIV-1 infection. J Immunol 171: 3837-3846.

S26. Frahm N, Baker B, Brander C (2008) Identification and optimal definition of HIV-derived cytotoxic T lymphocyte (CTL) epitopes for the study of CTL escape, functional avidity and viral evolution. Los Alamos HIV molecular immunology database.

S27. Janeway CA, Travers P, Walport M, Shlomchik MJ (2004) Immunobiology: Garland Science Publishing.

S28. Fideli US, Allen SA, Musonda R, Trask S, Hahn BH, et al. (2001) Virologic and immunologic determinants of heterosexual transmission of human immunodeficiency virus type 1 in Africa. AIDS Res Hum Retroviruses 17: 901-910.

S29. Operskalski EA, Stram DO, Busch MP, Huang W, Harris M, et al. (1997) Role of viral load in heterosexual transmission of human immunodeficiency virus type 1 by blood transfusion recipients. Transfusion Safety Study Group. Am J Epidemiol 146: 655-661.
