# Supplementary material for: Modelling the Evolution and Spread of HIV Immune Escape Mutants
Source: PLoS Pathog. 2010 Nov 18;6(11):e1001196. doi: 10.1371/journal.ppat.1001196 (PMC2987822; doi:10.1371/journal.ppat.1001196)
Supplement: Text S3 — A detailed analysis describing how additional factors could affect the evolution of escape mutants and thus affect our inferred rates of escape and reversion rates from the cross-sectional data (dataset 2). (0.41 MB PDF) [file ppat.1001196.s007.pdf]

Text S3. A detailed analysis describing how additional factors could affect the evolution of escape mutants and thus affect our inferred rates of escape and reversion rates from the cross-sectional data (dataset 2).

Factor 1. Restriction of our analysis to known escape mutants

Here we investigate whether restriction of our analysis to described escape mutants could have biased our inferred escape and reversion rates. In this section for each epitope included in the previous analysis we have calculated the proportion of HLA matched and HLA mismatched hosts with *any mutant* away from the B-clade ancestral sequence [S24]. Some of these mutants will be described escape mutants, some will be undescribed escape mutants and the remainder will be mutants that do not confer escape in the epitope. For each epitope with described escape mutants, Fig. S4 shows a direct comparison between the prevalence of *mutants* in the epitope (blue dots) and the prevalence of *described escape mutants* in the epitope (red dots). Two observations are noteworthy. The first is that if, at any given epitope escape typically occurs within, say, a year of infection we would expect to find a high prevalence of escape in hosts who are HLA matched for that epitope, i.e. the corresponding blue dot would lie to the far right of the plot. Since the majority of the blue dots do not lie to the far right of the plot, it is clear that for most epitopes escape does not typically occur within a year of infection. The second is that if at any particular epitope we have ruled out one or more mutations that confers escape we would expect the difference between the mutant prevalence and the genuine escape mutant prevalence to be higher in HLA matched hosts than in HLA mismatched hosts. This is because additional escape mutants would be more prevalent in HLA matched than -mismatched hosts. In comparing the *any mutant* prevalence to the *defined escape mutant* prevalence, we would expect to see some signal of this pattern, i.e. the gradient of the connecting line would be markedly shallower than the line $y=x$. For the majority of epitopes this is not what we see (Fig. S4). Thus for most of these epitopes it is unlikely that we have ruled out common escape mutants and underestimated the rate of escape.

Next we tested whether the epitopes that we have been investigating – those with previously described escape mutations in gag, RT and nef – are representative of all epitopes in these genes. Fig. S5 shows the prevalence of mutants away from the B-clade ancestral sequences in HLA matched and -mismatched hosts for 77 epitopes in gag, RT and nef. The list of epitopes used for this analysis is based upon the list of optimal epitopes provided in the Los Alamos Database [S26]. However, only epitopes restricted by HLA class I A and B alleles in regions of gag, RT and nef for which we had sequence data were included. In addition, epitopes with fewer than 3 HLA matched hosts and epitopes that were not defined as matching the B-clade ancestral sequence were excluded. Finally, epitopes with previously defined escape mutants (Table S1) were included in the list, even if they were not defined as optimal epitopes. Epitopes with known escape mutants (n=26) are displayed in red and the remaining epitopes (n=48) are displayed in green.

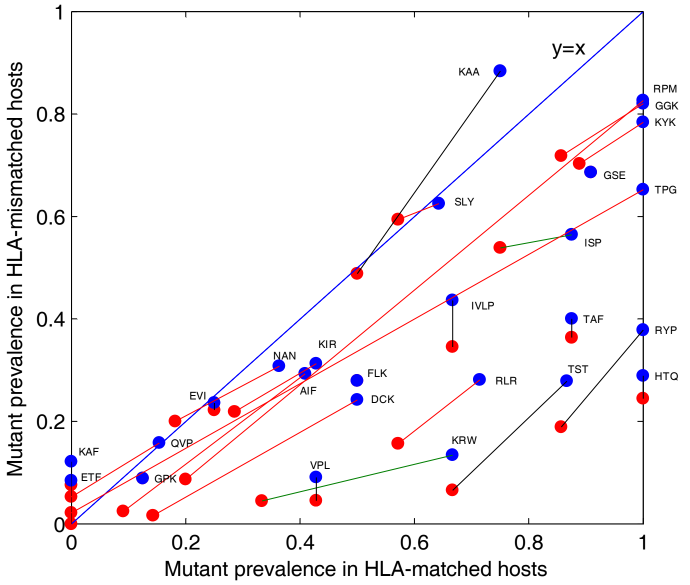

Figure S4. A scatter plot showing the prevalence of mutants (blue dots) and described escape mutants (red dots) in epitopes with described escape mutants in gag, RT and nef.

Lines connect the two data points for each epitope (n=26). Green lines (2 epitopes) represent epitopes for which the difference between the mutant prevalence and described escape mutant prevalence in HLA matched hosts is markedly larger than the equivalent difference in prevalence in HLA mismatched hosts. For these two epitopes there is evidence that by restricting our analysis to described escape mutations we have ignored enough genuine escape mutants to underestimate the rate of escape. Red lines (11 epitopes) represent epitopes for which the difference between the prevalence of mutants and defined escape mutants is only marginally greater in HLA matched than -mismatched hosts. For these epitopes we have probably ruled out a few uncommon escape mutants and very marginally underestimated the escape rate. Black lines (or no lines, 13 epitopes) connect the data points for all other epitopes. For these epitopes there is no evidence that we have ruled out genuine escape mutations.

The figure shows that all of the epitopes without described escape mutants lie close to the line $y=x$ and are distributed approximately evenly either side of that line. None of them lie towards the bottom right of the plot. Thus, there is little evidence of positive selection of escape mutants in these epitopes, suggesting that, if anything, escape rates amongst these epitopes are slower than amongst epitopes with described escape mutations.

To determine upper bounds on the escape rates we used the mathematical model to determine rates from the *mutant* prevalence data. Amongst the 26 epitopes with previously defined escape mutations this change in definition reduces the inferred time to escape from a median of 8.0 years to a median of 2.7 years (IQR=0.6-14.3 years). Across all optimally defined CTL epitopes escape is even slower (median=12.0 years). When this loosest definition of escape is applied to the data from the longitudinal cohort study (dataset 4), the inferred escape rates are no faster (Table S5).

Together, these analyses confirm that restriction of our analysis to defined escape mutants has not led to marked underestimation of our escape rates.

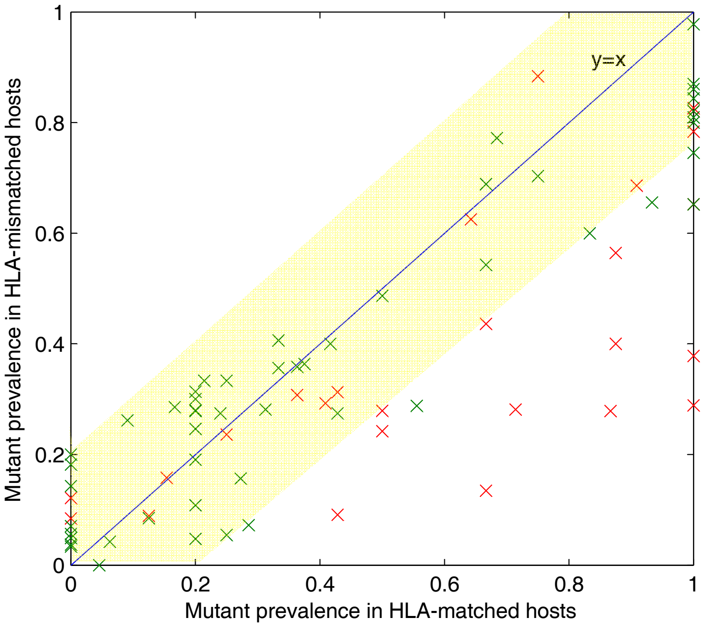

Figure S5. A scatter plot of mutant prevalence in hosts who are HLA matched and HLA mismatched for optimal CTL epitopes in gag, RT and nef.
The red crosses (n=26) represent epitopes in which escape mutants have been described and the green crosses (n=48) represent epitopes in which no known escape mutants have been described. This figure shows that all of the epitopes without described escape mutants lie close to the line $y=x$ and are distributed approximately evenly either side of that line, i.e. within the yellow box. None of them lie towards the bottom right of the plot. There is thus little evidence of selection of escape mutants in these epitopes.

Set of epitopes	Data source	Definition of escape mutant	Time to escape in HLA matched hosts (years)			Time to reversion in HLA mismatched hosts (years)		
			Median	IQR	No of epitopes with data	Median	IQR	No. of epitopes with data
Epitopes with defined escape mutants in gag, RT and nef	Cross-sectional data	Any mutation at a previously defined escape-site	8.0	1.8-34.0	26	No rev	6.5-No rev	25
	Longitudinal cohort		5.6	2.1-29.5	26	36.4	13.0-No rev	27
	Cross-sectional data	Any mutation in the epitope	2.7	0.6-14.3	26	No rev	44.4-No rev	26
	Longitudinal cohort		10.7	4.7-(>50)	26	120.0	42.5-(>50)	27
All optimally-defined epitopes in gag, RT and nef	Cross-sectional data		12.0	1.6-46.8	74	No rev	(>50)-No rev	74
	Longitudinal cohort		44.5	10.5-No esc	74	No rev	46.5-No rev	74

Table S5. Median and interquartile range (IQR) of escape and reversion rates derived from the cross-sectional data (dataset 2) and the longitudinal cohort (dataset 4). Two definitions of escape and two sets of escape mutants are considered.
It is noteworthy that analysis of the emergence of all mutations in longitudinal cohort study unexpectedly yields a larger median time to escape compared to the analysis of defined escape mutations. This is because the number of hosts with the wildtype strain at the first sample is not necessarily the same under these two definitions.

Factor 2. Reversion of escape mutants in HLA matched hosts

In the model described in the main text we made the simplification that an escape mutant would persist indefinitely in HLA matched hosts, either upon transmission of that mutant or following within-host selection of that mutant. It is possible, however, that continuous selection and reversion of escape mutations can take place within HLA matched hosts (Fig. S6). These types of dynamics are most probable in the setting of escape mutants that affect the recognition of the epitope by the T-cell-receptor (TCR). Such mutants have the potential to stimulate a new population of CTLs with different TCRs that can recognize the mutated epitope. This could happen either upon transmission to a new HLA matched host or later on in the same host. Recognition of the mutant epitope would eliminate any selective advantage the mutant previously had and the virus population could revert to wildtype if the mutant has an associated fitness cost. Alternatively, or at a later time, a new escape mutant could be selected for in place of the original mutant[14], and this process may happen again and again, possibly with the original mutant being reselected at some stage.

To understand how the prevalence of an escape mutant in the population would be affected by reversion in HLA matched hosts we have adapted the original model to allow reversion – as well as escape – to take place in HLA matched hosts. Reversion in HLA matched hosts is assumed to occur at rate $\tilde{\psi}$ (Fig. S7).

This model predicts that the prevalence of escape in HLA matched and HLA mismatched hosts would be lower if reversion occurs in HLA matched hosts (Fig. S8). This is because mutants will persist for shorter periods of time in HLA matched hosts. On average, HLA matched hosts will also transmit escape mutants less frequently; hence the prevalence of escape in HLA mismatched hosts will also be lower.

This model can be used to estimate the rate of escape in HLA matched hosts and the rate of reversion in HLA mismatched from cross-sectional escape prevalence data. To perform this calculation the rate of reversion in HLA matched hosts needs to be estimated and entered as a parameter into the model.

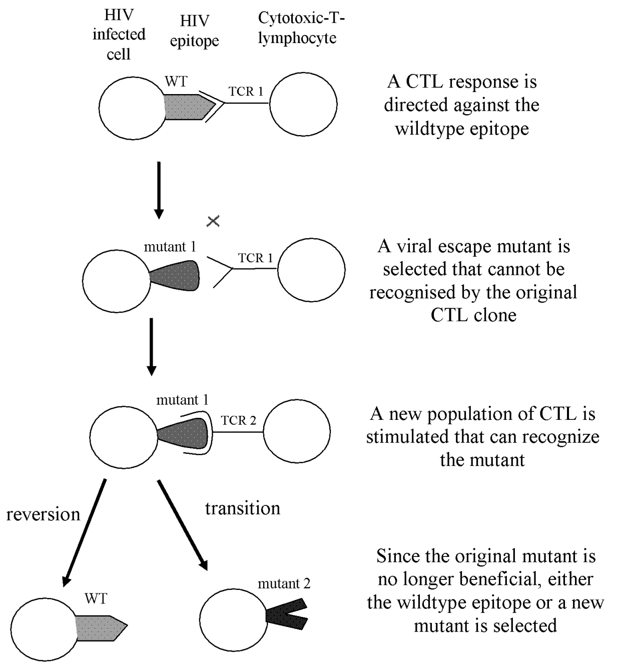

Figure S6. A diagram describing how reversion of CTL escape mutants and transitions between escape mutants could occur in HLA matched hosts.

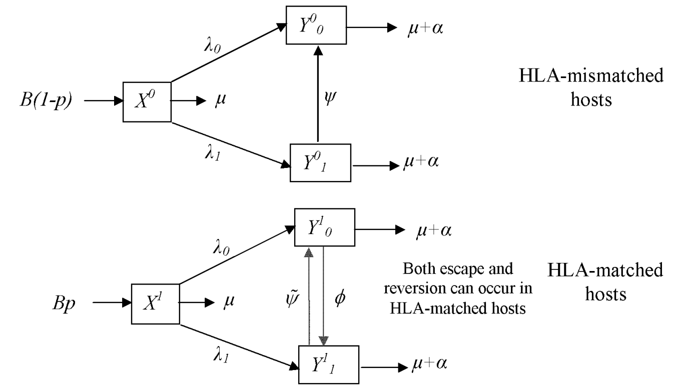

$$\frac{dX^h}{dt} = \left(\delta_{h0}(1-p)+\delta_{h1}p\right)B - (\lambda_0+\lambda_1+\mu)X^h$$

$$\frac{dY_v^h}{dt} = \lambda_v X^h + (2\delta_{hv}-1)\left(\delta_{h1}\left(\phi Y_0^1 - \tilde{\psi} Y_1^1\right)+\delta_{h0}\psi Y_1^0\right) - (\mu+\alpha)Y_v^h$$

$$\lambda_v = \beta c\left(\sum_{h=0,1} Y_v^h\right) \Big/ \sum_{h=0,1}\left(X^h + \sum_{v=0,1} Y_v^h\right)$$

Figure S7. A schematic diagram and ordinary differential equations describing a model of selection, reversion and transmission of CTL escape mutants that includes both escape and reversion of mutants in HLA matched hosts. In these equations and throughout this section δ_{ij} represents the Kronecker delta (1 if i=j and 0 if i≠j).

Using this method it can be shown that inferred escape rates in HLA matched hosts would be faster under the assumption that reversion occurs in HLA matched hosts. For example, if the average time to reversion in HLA matched hosts were 10 years, the time to escape inferred from the Swiss cross-sectional data reduces from a median of 8.0 years to a median of 4.5 years (IQR=1.0-18.7 years). The inferred reversion rates in HLA mismatched hosts hardly change at all.

It is not possible to estimate reversion rates in HLA matched hosts from the cross-sectional data. However, data from the longitudinal cohort study (dataset 4) shows that across all epitopes with described escape mutations for which data were available (n=26) there were an average of only 1 reversion per 25 person-years of observation. At this rate ($\tilde{\psi} = 1/25$ years^{-1}) reversion in HLA matched hosts would not substantially affect the inferred rates (Fig. S9).

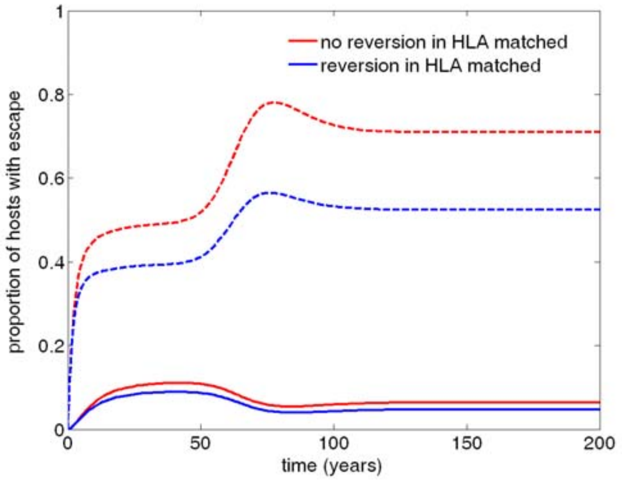

Figure S8. The prevalence of escape in HLA matched (dashed lines) and HLA mismatched (solid lines) hosts would be lower if reversion occurs in HLA matched hosts.
In this graph red lines represent the scenario where there is no reversion in HLA matched hosts ($\tilde{\psi} = 0$ years^{-1}) and blue lines represent the scenario when there is reversion in HLA matched hosts ($\tilde{\psi} = 1/10$ years^{-1}). The remaining model parameters and starting values used for this figure are as follows: $\phi = 1/5$, $\psi = 1/10$, $p = 0.1$, $\mu = 1/50$, $\mu + \alpha = 1/10$, $\beta c = 0.3$, $B = 10^5 \mu$, $X^0(0) = 9 \times 10^4$, $X^1(0) = 10^4$, $Y_0^0(0) = 0.9$, $Y_0^1(0) = 0.1$ and $Y_1^1(0) = Y_1^0(0) = 0$.

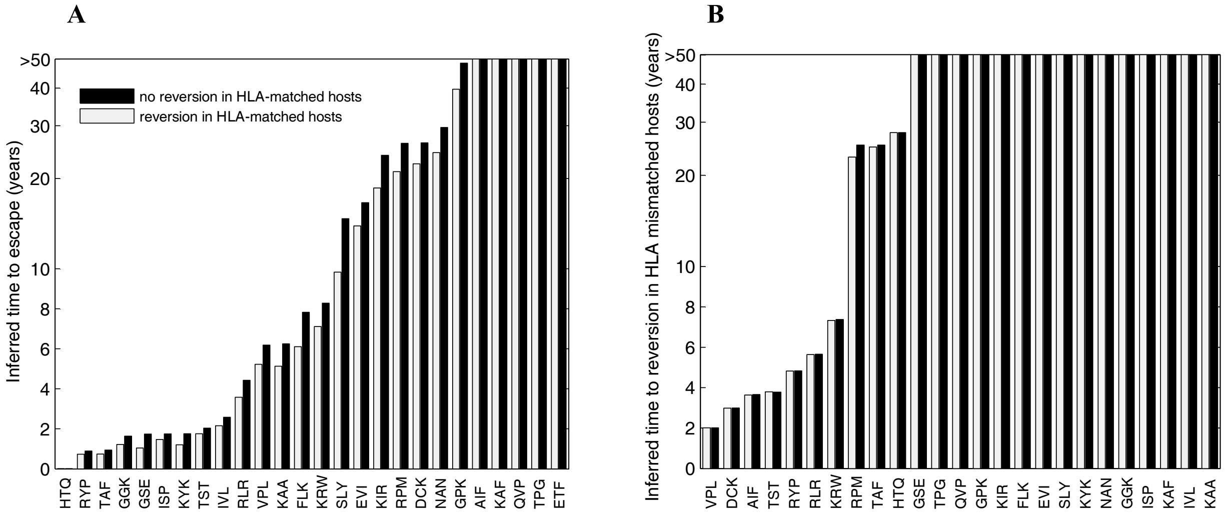

Figure S9. Rates of escape in HLA matched hosts (A) and reversion in HLA mismatched hosts (B) inferred from the Swiss cross-sectional data under the assumption that reversion occurs in HLA matched hosts.
The black bars represent the inferred rates under the assumption that there is no reversion in HLA matched hosts ($\tilde{\psi}$=0 years^{-1}). The white bars represent the inferred rates under the assumption that reversion occurs in HLA matched hosts at a rate of one reversion per 25 person-years of observation ($\tilde{\psi}$=1/25 years^{-1}), as estimated from the longitudinal cohort study (dataset 4). These figures show that if reversion in HLA matched hosts is as slow as estimated, the original model would lead to only marginal underestimation of the escape rates. Reversion rates change very little indeed (in both directions). The remaining parameters used for these estimations are as follows: μ+α=1/10, βc=0.3 (thus R_0=3) and t=*27* years.

Factor 3. Transitions between different escape mutants

As described in the previous section and Fig. S6 it is possible that one escape mutant can be selected in place of another escape mutant in HLA matched hosts, i.e. transitions can occur between different escape mutants.

To investigate how this could affect the evolution of escape mutants and thus affect our escape and reversion rate estimates we have made an extension to the model presented in Fig. S7 in which two different, mutually exclusive escape mutants can be selected at the same epitope (Fig. S10). In this model different escape mutants are denoted as virus variants v=1 and v=2. Selection from the wildtype (v=0) to escape variant v occurs at rate ϕ_{0v}. Reversion from escape mutant v to the wildtype occurs at rate $\tilde{\psi}_{v0}$ in HLA matched hosts and at rate ψ_{v0} in HLA mismatched hosts. Transitions from escape mutant 1 to escape mutant 2 occur at rate ϕ_{12} and transitions in the opposite direction occur at rate ϕ_{21}.

The rates that we have estimated from the cross-sectional data represent the rates at which HLA matched hosts infected with the wildtype virus can select an escape mutant. These estimates are based upon the prevalence of escape mutants in the population. Any single transition would not directly affect the prevalence of escape in the population. From a broader perspective, transitions would also have no effect at all on the escape prevalence if the different escape mutants revert at the same rate. This can be shown using model simulations (Fig. S11). It can be explained by considering the model shown in Fig. S10 under the restrictions that the reversion rates of the two mutants in HLA matched hosts are both equal to $\tilde{\psi}$ ($\tilde{\psi}_1 = \tilde{\psi}_2 = \tilde{\psi}$) and the reversion rates of the two mutants in HLA mismatched hosts are both equal to ψ ($\psi_1 = \psi_2 = \psi$). In this particular case, the model in Fig. S10 collapses back into the model presented in Fig. S5 – which is independent of transition rates – but with an overall escape rate equal to sum of the escape rates of the two mutants ($\phi = \phi_1 + \phi_2$).

If transitions are rapid and the reversion rates of different escape mutants at the same epitope are markedly different,

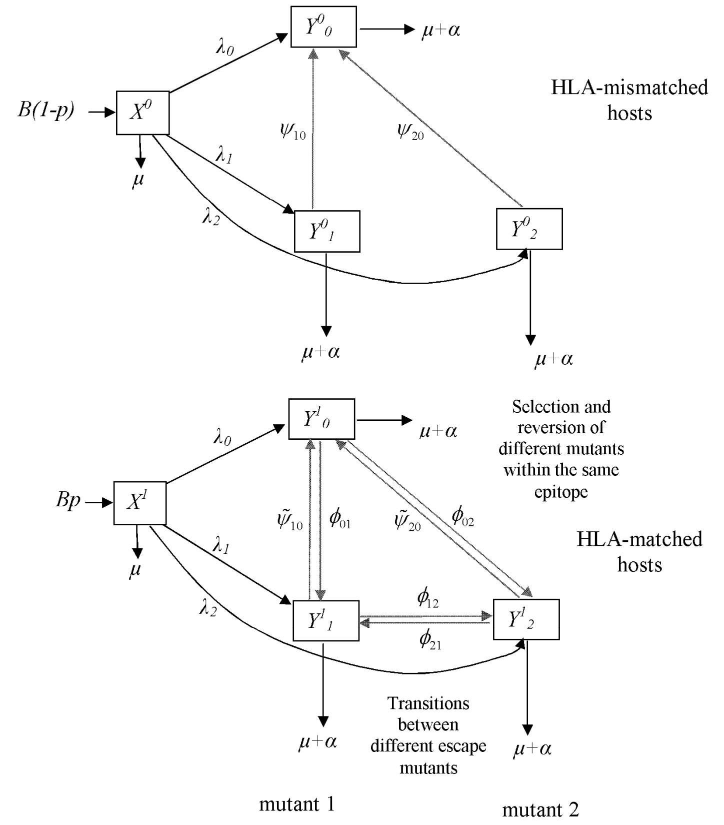

$$\frac{dX^h}{dt} = \left(\delta_{h0}(1-p) + \delta_{h1}p\right)B - \left(\mu + \sum_{v=0,1,2} \lambda_0\right)X^h$$

$$\frac{dY^h_v}{dt} = \lambda_v X^h - (\mu+\alpha)Y^h_v + (\delta_{v1} - \delta_{v0})\left(\delta_{h1}(\phi_{01}Y^1_0 - \tilde{\psi}_{10}Y^1_1) + \delta_{h0}\psi_{10}Y^0_1\right)$$

$$+(\delta_{v2} - \delta_{v0})\left(\delta_{h1}(\phi_{02}Y^1_0 - \tilde{\psi}_{20}Y^1_2) + \delta_{h0}\psi_{20}Y^0_2\right) + (\delta_{v1} - \delta_{v2})(\phi_{12}Y^1_1 - \phi_{21}Y^1_2)$$

$$\lambda_v = \beta c\left(\sum_{h=0,1} Y^h_v\right) \Bigg/ \sum_{h=0,1}\left(X^h + \sum_{v=0,1} Y^h_v\right)$$

Figure S10. A schematic diagram and ordinary different equations describing a model of selection, reversion and transmission of CTL escape mutants that includes both escape and reversion of mutants in HLA matched hosts, and transitions between different mutants that confer escape for the same epitope.

transitions can affect escape prevalences. Model simulations with plausible parameter values, however, suggest that escape prevalences would not vary substantially. Of note, it seems probable that the rate at which any particular escape mutant emerges in place of another mutant – i.e. through transitions – would be no faster than the rate at which that mutant emerges in place of the wildtype strain ($\phi_{21} \le \phi_{01}$ and $\phi_{12} \le \phi_{02}$). If we add in the assumption that for each escape mutant, these two rates are equal ($\phi_{21} = \phi_{01}$ and $\phi_{22} = \phi_{02}$) transitions have very little impact indeed on the evolution of escape, irrespective of the other parameter values. This result holds if the transition rates are slower than these rates, i.e. if $\phi_{21} \le \phi_{01}$ and $\phi_{12} \le \phi_{02}$.

In summary, transitions between different escape mutants at any particular epitope would not substantially affect the escape prevalence at that epitope. Transitions would therefore not substantially affect our inferred rates of escape or reversion.

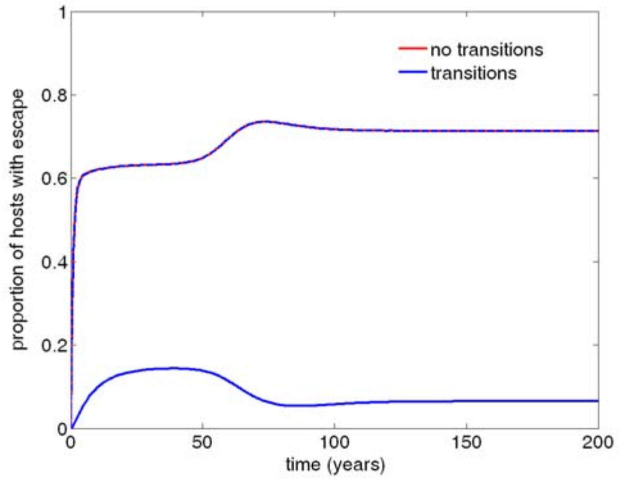

Figure S11. Transitions between different escape mutants within any particular epitope would not substantially affect the prevalence of escape in HLA matched (dashed lines) or HLA mismatched (solid lines) hosts at that epitope.

This figure shows how evolution would be affected by transitions under the assumption that different mutants within the same epitope revert at the same rates – i.e. $\psi_{10} = \psi_{20}$ and $\tilde{\psi}_{10} = \tilde{\psi}_{20}$. Red lines represent the case when there is no transitions between mutants ($\phi_{12} = \phi_{21} = 0$) and the overlapping blue lines represent the case when there are transitions ($\phi_{21} = 1/5$ and $\phi_{12} = 1/2$). This shows that under the assumption of equal reversion rates, transitions between mutants would not affect the evolution of escape mutants at all. The remaining model parameters and the starting values used in this figure are: $\phi_{01} = 1/5$, $\phi_{02} = 1/2$, $\psi_{10} = \psi_{20} = 1/10$, $\tilde{\psi}_{10} = \tilde{\psi}_{20} = 1/5$, $p = 0.1$, μ=1/50, $\mu+\alpha$=1/10 $\beta c = 0.3$, $B = 10^5 \mu$ $X^0(0) = 9\times10^4$, $X^1(0) = 10^4$, $Y_0^0(0) = 0.9$, $Y_0^1(0) = 0.1$ and $Y_1^1(0) = Y_1^0(0) = Y_2^1(0) = Y_2^0(0) = 0$.

Factor 4: Faster escape and/or reversion early on during infection

In Fig. 2C and 2D we presented data on CTL escape and reversion events observed in case reports of individual patients. The vast majority of the escape and reversion events that were observed (40/45 escape events and 3/3 reversion events) occurred during the first 3 years of infection. Given that untreated persons survive on average 10 years once infected with HIV [39], a disproportionate number of the observed escape and reversion events in the case reports thus occurred early on during infection. This was most likely, at least in part, caused by the fact that many of the studies followed patients for only a few years, rather than until the end of their infection. However, it may also have been caused partly by genuinely faster escape and reversion rates during early infection compared to later infection.

Here we make an adaptation to the original model to include faster escape and/or faster reversion earlier on during infection (i.e. during the first year or two of infection). A schematic diagram of this model is presented in Fig. S12. In this model the four infected groups in the original model, Y_v^h, are each subdivided into two groups representing hosts who are in the early stages of infection, U_v^h, and hosts who have been infected for a longer period of time, Z_v^h. When susceptible hosts become infected they first enter the 'early infection' state. At a rate τ these hosts move into the corresponding 'late infection' state. The death rate of both uninfected hosts and hosts in early infection is μ and the death rate of hosts in late infection is $\mu+\alpha$. Escape in HLA matched hosts occurs at rate ϕ_1 during early infection and ϕ_2 ($\leq \phi_1$) during late infection. Reversion in HLA mismatched hosts occurs at rate ψ_1 during early infection and ψ_2 ($\leq \psi_1$) during late infection. Using this model we find that the escape prevalences in both HLA matched and HLA mismatched hosts increase as the rate of escape in both infection stages (ϕ_1 and ϕ_2) increase (Fig. S13). Equivalently, if escape is faster during early infection, the average escape rate across the full period of an infection will be slower than the escape rate during early infection. This means that the rate of escape during early infection would be underestimated from cross sectional data under the assumption of homogeneous rates. An extreme example of heterogeneous rates would be if no escape occurs beyond 1 year of infection. Under this assumption ($\phi_2 = 0$, $\psi_1 = \psi_2 > 0$, $\tau = 1$) the average number of person-years of observation per escape during the first year of infection inferred from the cross sectional data reduces from a median of 8.0 years to a median of 2.0 years^{-1} (IQR: 0.5-7.0 years^{-1}).

A similar argument shows that if reversion is faster earlier on during infection, reversion rates during early infection would be underestimated under the assumption of homogeneous rates. If the average number of person-years per observation per reversion during the first year of infection are estimated from the cross sectional data under the assumption that no reversion occurs beyond 1 year (ψ_2=0, $\phi_1 = \phi_2 > 0$, $\tau = 1$), they decrease from a median of 60.0 years to a median of 12.5 years. If it is assumed that neither escape nor reversion occur beyond 1 year ($\psi_1 = \psi_2 = 0$) the results are very similar. Escape and reversion rates at different stages post infection have been measured directly from the longitudinal cohort study (Fig. S14).

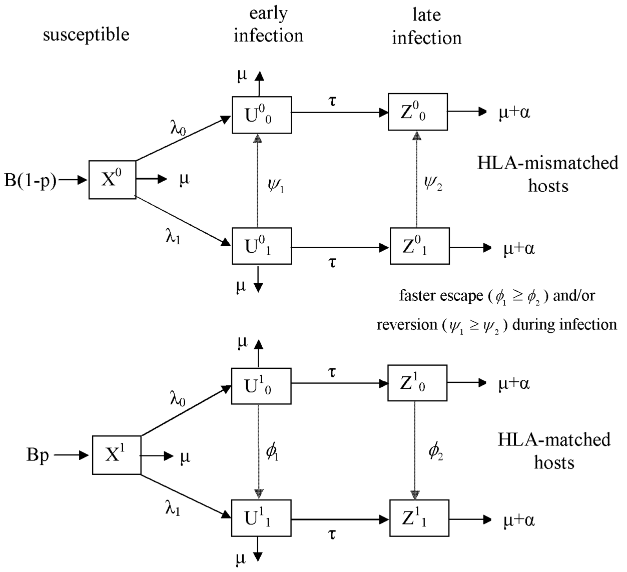

$$\frac{dX^h}{dt} = \left(\delta_{h0}(1-p) + \delta_{h1}p\right)B - (\lambda_0 + \lambda_1 + \mu)X^h$$

$$\frac{dU_v^h}{dt} = \lambda_v X^h + (2\delta_{hv} - 1)\left(\delta_{h1}\phi_1 U_0^1 + \delta_{h0}\psi_1 U_1^0\right) - (\mu + \tau)U_v^h$$

$$\frac{dZ_v^h}{dt} = \tau U_v^h + (2\delta_{hv} - 1)\left(\delta_{h1}\phi_2 Z_0^1 + \delta_{h0}\psi_2 Z_1^0\right) - (\mu + \alpha)Z_v^h$$

$$\lambda_v = \beta c\left(\sum_h \left(U_v^h + Z_v^h\right)\right) \Big/ \sum_h \left(X^h + \sum_v \left(U_v^h + Z_v^h\right)\right)$$

Figure S12. A schematic diagram and ordinary differential equations describing a model of selection, reversion and transmission of CTL escape mutants that includes more rapid evolution early on during infection, compared to later on during infection.

These observations suggest that the heterogeneity in rates is not nearly as extreme as the example described above. The escape rate is approximately 1.6 fold faster during the first year of infection than beyond the first year. The reversion rate is 1.8 fold faster during the first 3 years of infection. These small changes suggest that differential transmission rates would have very little effect indeed upon the escape and reversion rates inferred from the cross-sectional data. This is shown in Fig. S15 which shows the inferred rates under the assumption that both escape and reversion are twice as fast during the first year of infection than beyond the first year. The average number of person-years of observation per escape during the first year of infection drops from a median of 8.0 years to a median of 5.1 years (IQR= 1.3-21.0 years). It is noteworthy, however that individuals enrolled onto the longitudinal cohort study provided their first sample a median of six weeks following their estimated date of infection. Whether escape and/or reversion rates are substantially faster during the first few weeks of infection therefore remains an open question. If they are, it would affect this result.

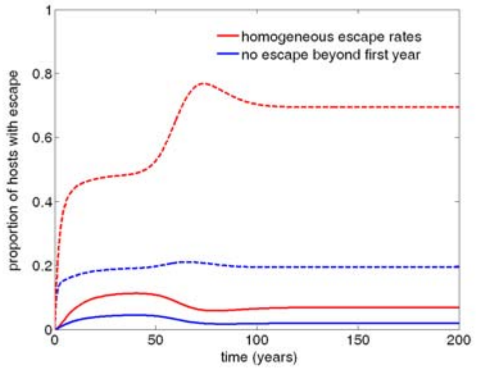

Figure S13. The escape prevalence in HLA matched (dashed lines) and mismatched hosts (solid lines) would be substantially lower under the assumption that is no escape occurs beyond the first year of infection.
Escape prevalences are shown in red when escape rates are homogeneous with respect to time since infection ($\phi_1 = \phi_2 = 1/5$) and in blue when there is no escape beyond 1 year ($\phi_1 = 1/5$, $\phi_2 = 0$). The remaining model parameters and starting values used for these plots are: $\psi_1 = \psi_2 = 1/10$, $p = 0.1$, $\tau = 1$, $\mu = 1/50$, $\mu + \alpha = 1/9$, $\beta c = 0.3$, $B = 10^5 \mu$, $X^0(0) = 9\times10^4$, $X^1(0) = 10^4$, $U_0^0(0) = 0.9$, $U_0^1(0) = 0.1$ and $U_1^1(0) = U_1^0(0) = Z_v^h(0) = 0 \;\; \forall (h,v)$.

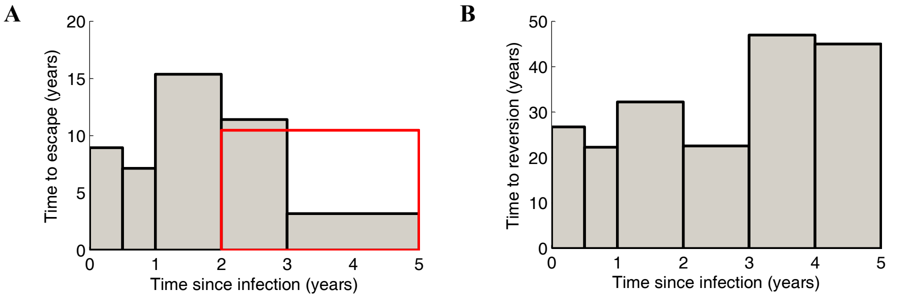

Figure S14. Bar charts showing observed changes in the rate of escape (A) and rate of reversion (B) over the course of an infection. Data from the longitudinal cohort study (dataset 4). In this analysis data from 27 epitopes with described escape mutants in gag, RT and nef are grouped together. The red bar in panel A) shows the result of grouping together data from years 2-5. These data indicate that escape is approximately 1.6 fold faster during the first year of infection than beyond the first year. Reversion is approximately 1.8 fold faster during the first 3 years than beyond the first 3 years.

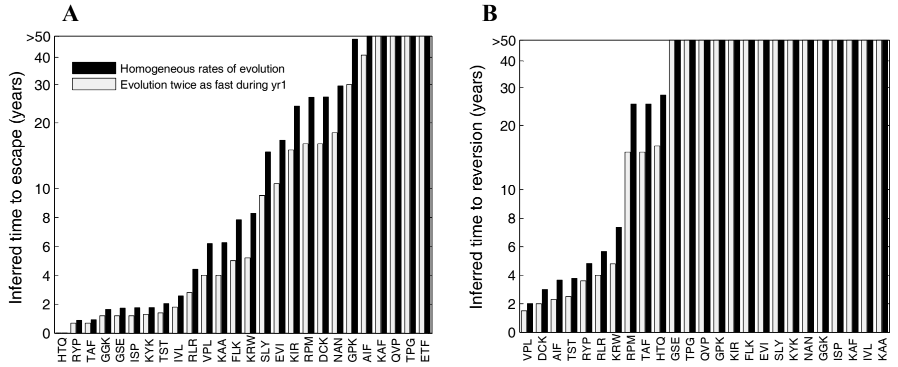

Figure S15. If escape and reversion were twice as fast during the first year of infection than beyond the first year of infection it would not have any serious effect upon our inferred escape and reversion rates during the first year of infection
Inferred escape (A) and reversion (B) rates during the first year of infection derived from the Swiss cross-sectional data. This figure compares inferred rates based upon two different assumptions. Firstly that evolution occurs at the same rate throughout infection (red bars; $\phi_1 = \phi_2$ and $\psi_1 = \psi_2$) and secondly that evolution is twice as fast during early infection compared to late infection (blue bars, $\phi_1 = 2\phi_2$, $\psi_1 = 2\psi_2$). All model estimates assume that $\tau = 1$, $\mu + \alpha = 1/9$, $\beta c = 0.3$ and $t = 27$.

Factor 5. Differential transmission rates at different stages of infection (i.e. more rapid transmission during acute infection)

Viral loads are typically higher during acute infection and late-stage infection than during chronic infection [S27] and a positive correlation has been found between viral load and the probability of transmission [S28,S29]. It should follow that the probability of transmission per unprotected coital act is higher for hosts in acute and late-stage infection. Evidence in favour of this comes from a study of HIV-discordant heterosexual pairs [40] which showed that transmission per coital act during the first 2.5 months after seroconversion was 10 times more likely than during the chronic phase. Transmission during the last 2 years before death was 4.6 times more likely per coital act compared to the chronic phase, though hosts in late-stage infection also reported that they had sex two thirds as often as patients in primary or chronic infection. Overall, these data suggest that compared to chronic infection, transmission is 10 times and 3 times more likely in acute infection and late-stage infection, respectively.

In this section we make adaptations to the original model to understand how the evolution of CTL escape mutants, and thus our inferred escape and reversion rate estimates, would be affected by faster transmission rates during acute infection compared to chronic infection. For simplicity, we do not explicitly model differential transmission rates during late-stage infection, though we do assess the impact of this factor heuristically.

A schematic diagram of the model used for this analysis is presented in Fig. S16. This is very similar to the model presented in the Fig. S12 because it models the dynamics of hosts in early and late infection separately. In this model, however, early infection represents acute infection – assumed here to be the first 2.5 months of infection. We also assume that newly infected patients are more infectious than chronically infected patients. They contribute to the force of infection in a ratio of $\beta_U c_U$ to $\beta_Z c_Z$. Finally, because CTL responses are not fully established during acute infection we assume that escape does not occur during acute infection. Instead, we assume that reversion of escape mutants can occur in HLA matched hosts during

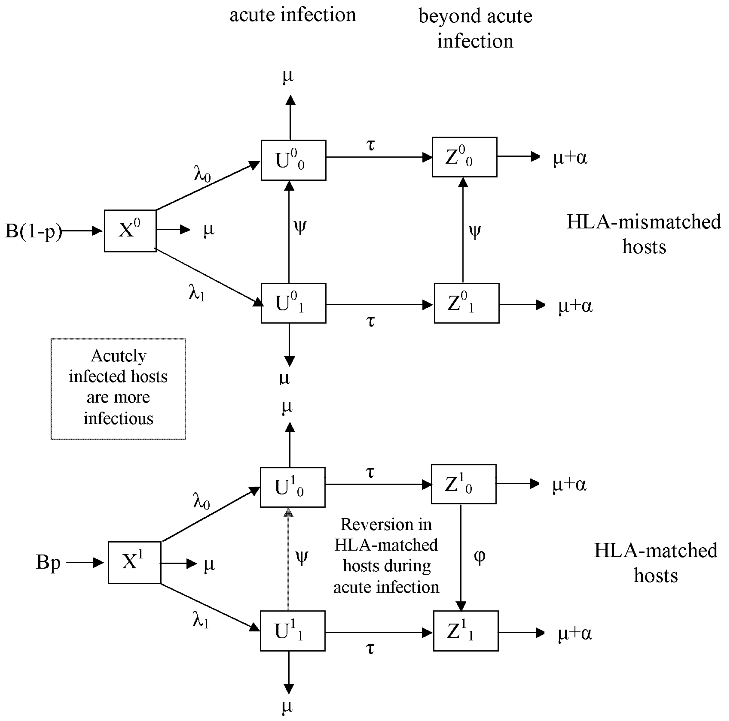

$$\frac{dX^h}{dt} = \left(\delta_{h0}(1-p) + \delta_{h1}p\right)B - (\lambda_0 + \lambda_1 + \mu)X^h$$

$$\frac{dU_v^h}{dt} = \lambda_v X^h + (2\delta_{v0} - 1)\psi U_1^h - (\mu + \tau)U_v^h$$

$$\frac{dZ_v^h}{dt} = \tau U_v^h + (2\delta_{hv} - 1)\left(\delta_{h1}\phi Z_0^1 + \delta_{h0}\psi Z_1^0\right) - (\mu + \alpha)Z_v^h$$

$$\lambda_v = \left(\sum_h \left(\beta_U c_U U_v^h + \beta_Z c_Z Z_v^h\right)\right) \Big/ \sum_h \left(X^h + \sum_v \left(U_v^h + Z_v^h\right)\right)$$

Figure S16. A schematic diagram and ordinary differential equations describing a model of selection and reversion of CTL escape mutants that includes faster transmission from hosts in acute infection compared to hosts in chronic infection. Reversion of CTL escape mutants occurs in both HLA matched and -mismatched hosts during acute infection.

acute infection. In Fig. S17 we consider escape prevalences in two model epidemics that have the same dynamics in terms of early epidemic growth. In blue the transmission coefficient for hosts infected for less than 2.5 months (acute infection) is 10 times higher than it is for hosts infected for more than 2.5 months ($\beta_U c_U = 10\beta_Z c_Z$). In red, the transmission rate is the same for hosts in both infection stages ($\beta_U c_U = \beta_Z c_Z$). This figure shows that realistic differential transmission rates during acute and chronic infection would have very little impact upon the prevalence of escape mutants in the population. The main reason for this is that even if transmission is 10 times faster during acute infection, acutely infected hosts still account for a minority of all infections because the duration of acute infection is short compared to the whole duration of infection – approximately 2.5 months [40] compared to approximately 10 years in untreated hosts [39]. Indeed, it has been estimated that only 15-20% of all infections can be attributed to acutely infected hosts [40, 41]. In addition to this, the observed prevalence of mutants in HLA matched hosts is highly dependent upon the within-host rate of escape, not just the fraction of transmitted variants that contain the mutant. This means that even in the extreme case when the vast majority of infections are from acutely infected hosts, the prevalence of escape mutants in HLA matched hosts will still be close to the prevalence expected under equal transmission rates, particularly if mutants revert rapidly in HLA mismatched hosts. Similar analysis (results and model not shown) reveals that if transmission is three times faster in late-stage infection compared to chronic infection it would also not noticeably affect the evolution of escape mutants at the population level.

In summary, plausible differences in the transmission rates at different stages during infection would not be sufficient to affect the evolution of escape mutants at the population level. Differential transmission rates would therefore not affect our escape and reversion rate estimates.

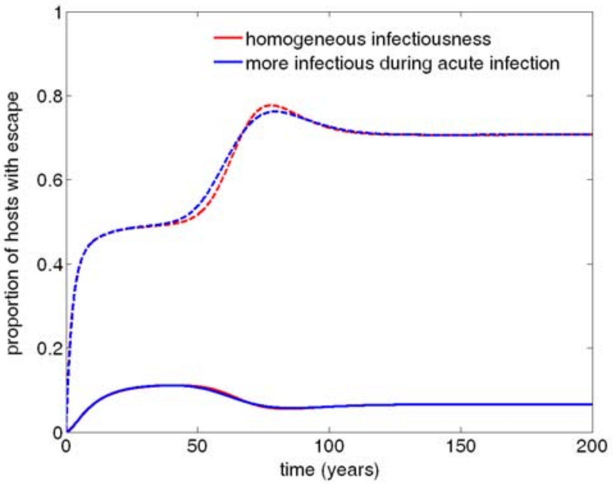

Figure S17. **The escape prevalence in HLA matched (dotted lines) and HLA mismatched hosts (solid lines) is largely invariant to faster transmission during acute infection.**
In this figure the red lines represent the escape prevalences under the assumption that transmission occurs at the same rate throughout infection ($\beta_U c_U = \beta_Z c_Z$). The blue lines represent the escape prevalences when transmission is 10 times faster during the first 2.5 months of infection ($\beta_U c_U = 10\beta_Z c_Z$) compared the rest of infection. The figure shows that faster transmission during acute infection does not substantially affect the escape prevalence in either host type. To ensure the same overall transmission rates and thus the same epidemic dynamics between the two scenarios we assumed that $\beta_Z c_Z$=0.3 for the constant transmission rate scenario and that $\beta_Z c_Z$=0.196 for the differential transmission rates scenario. Other model parameters and starting values that we used in these figures are: ϕ=1/5, ψ=1/10, $p = 0.1$, τ=1/0.2, $\mu = 1/50$, $\mu + \alpha = 1/9.8$, $B = 10^5 \mu$, $X^0(0) = 9\times10^4$, $X^1(0) = 10^4$, $U_0^0(0) = 0.9$, $U_0^1(0) = 0.1$ and $U_1^1(0) = U_1^0(0) = Z_v^h(0) = 0 \quad \forall(h,v)$.

Factor 6. Overlapping epitopes

A large proportion of CTL epitopes lie within overlapping sections of the genome and this could potentially affect the evolution of escape mutants at the population level. Restriction to two different epitopes may drive mutations which confer escape in both epitopes (Fig. S18). Alternatively, they may drive different mutations which confer escape only in the epitope for which the mutation was selected. These mutations may arise at the same site, or at different sites within the overlap of the two epitopes.

To investigate the effects of overlapping epitopes we have adapted our original model to represent the dynamics of a particular mutation at a single site that confers escape in two overlapping epitopes restricted by two different HLA alleles (Figs. S18 and S19). In this adaptation there are four host types (h_1h_2= 00, 10, 10, or 11) defining whether or not a host restricts each of the two epitopes. For example, h_1h_2=10 defines a host who restricts epitope 1, but not epitope 2. A proportion, $p^{h_1h_2}$, of the population are of each host type. As in the original model, there are two viral types – virus with the escape mutation (v=1) and virus the wildtype epitope (v=0). Selection of the escape mutation takes place in hosts who restrict at least one, or both of the epitopes at rates $\phi^{h_1h_2}$. The rate of escape in hosts who restrict both epitopes (ϕ^{11}) is assumed to be at least as fast as the faster of the two rates of escape in hosts who restrict only on or other of the two epitopes ($\phi^{11} \geq \max(\phi^{10}, \phi^{01})$). In hosts who don't restrict either of the two epitopes, reversion occurs at rate ψ. All other dynamics of the model are as described for the original model.

This model shows that the prevalence of the escape mutation in the population will be higher if it is being driven by two overlapping epitopes than if it is just being driven by one epitope and that the difference can be substantial. Escape prevalence in each of the four host types will increase if the proportion of hosts with each HLA restriction is larger and if the rates of escape in hosts who restrict each, and both, of these epitopes (ϕ^{10}, ϕ^{01} and ϕ^{11}) are faster. In Fig. S20 we

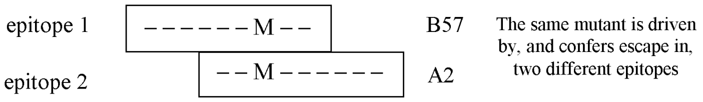

Figure S18. A diagram showing how the same escape mutation could be driven by the restriction to two different epitopes.

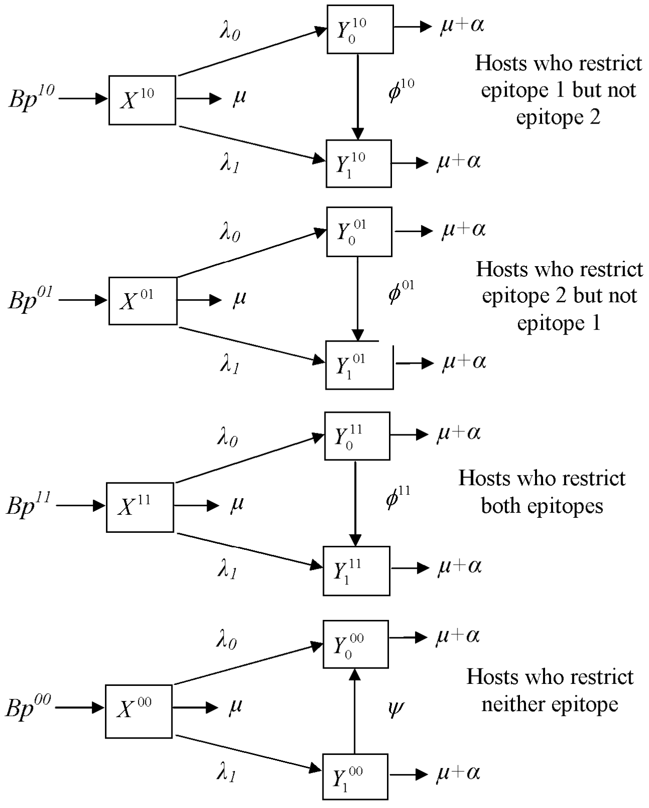

$$\frac{dX^{h_1h_2}}{dt} = Bp^{h_1h_2} - (\lambda_0 + \lambda_1 + \mu)X^{h_1h_2}$$

$$\frac{dY_v^{h_1h_2}}{dt} = \lambda_v X^{h_1h_2} + (1-2\delta_{v0})\Big((1-\delta_{h_10}\delta_{h_20})\phi^{h_1h_2}Y_0^{h_1h_2} - \delta_{h_10}\delta_{h_20}\psi Y_1^{00}\Big) - (\mu+\alpha)Y_v^{h_1h_2}$$

$$\lambda_v = \beta c\left(\sum_{h_1}\sum_{h_2} Y_v^{h_1h_2}\right)\Big/\sum_{h_1}\sum_{h_2}\left(X^{h_1h_2} + \sum_{v} Y_v^{h_1h_2}\right)$$

Figure S19. A schematic diagram and ordinary differential equations describing a model of selection and transmission of a CTL escape mutation that is driven by different HLA restrictions to two overlapping epitopes.

show, using this model, how escape prevalence data could be misinterpreted if it is assumed that a mutation at a particular epitope (epitope 1) is being driven only by the HLA restriction to that epitope, when in fact it is also being driven by a different HLA restriction to an overlapping epitope (epitope 2). In this situation, some hosts who are HLA mismatched for epitope 1, and assumed *not* to restrict the epitope, will be HLA matched for epitope 2 and will also be driving the escape mutation. As a result, the prevalence of escape in hosts who are HLA mismatched for epitope 1 can be much higher than expected. This effect will be greatest if the rate of escape in the overlapping epitope (epitope 2) is fast and if the proportion of the population that restricts the overlapping epitope is high.

In the model presented above, hosts who restrict overlapping epitopes work in unison to drive identical escape mutations that confer escape in both epitopes. Alternatively, restrictions to overlapping epitopes could drive different mutations that confer escape only in the epitope for which the mutant was selected. If such mutations lie within the same site then the evolutionary processes driven by the overlapping epitopes would counteract each other since each mutant would sometimes be selected in place of the other mutant. This would not have any impact – beyond that described above – upon our analysis of defined escape mutations because we have counted all possible mutations at previously defined escape sites as escape mutations.

If two mutations lie within different sites then evolution of each mutation would not be affected by evolution of the other mutation. This scenario would not have any impact upon our analysis restricted to mutations at previously defined escape sites. It would, however, lead to a higher than expected escape prevalence in the epitope when considering the '*any mutant'* prevalence.

In summary, for any particular epitope, overlapping epitopes act to increase the prevalence of mutants in hosts who are both HLA matched and HLA mismatched for the index epitope. A higher than expected escape prevalence in HLA matched hosts means that overlapping epitopes could therefore led us to underestimate reversion rates. Since overlapping epitopes would also drive an increase in the escape prevalence in HLA matched hosts, they would typically lead to overestimation, not underestimation of the escape rates. Overlapping epitopes therefore do not explain why the escape rates inferred from the cross-sectional data are slower than the rates inferred from the case reports.

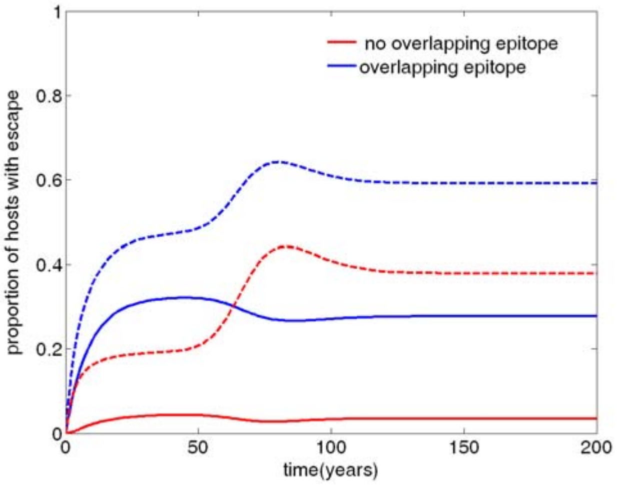

Figure S20. Plots showing how an escape mutant would evolve if it is being driven by hosts who restrict two overlapping epitopes.
This figure shows the escape prevalence in hosts who are HLA matched (dashed lines) and HLA mismatched (solid lines) for epitope 1 under two assumptions. Firstly assuming that the mutant is being driven only by hosts who restrict epitope 1 (red lines; original model with ϕ=1/20) and secondly assuming that the mutant is being driven by hosts who restrict both epitopes (blue lines; overlapping model with $\phi^{10} = 1/20$, $\phi^{01} = 1/5$ and $\phi^{11} = 1/4$). This shows that the escape prevalence in both host types is higher if the mutation is also being driven by hosts who restrict an overlapping epitope (epitope 2) than if it is just being driven by epitope 1. For simplicity, we have assumed that restriction of different epitopes is independent, i.e. $p^{10} = p^{1}(1-p^{2})$, where p^1=*0.1* and p^2=*0.2* represent the proportion of the population who restrict epitope 1 and epitope 2, respectively. The remaining model parameters and starting values used are: ψ=1/10, $\mu = 1/50$, $\mu + \alpha = 1/10$, $\beta c = 0.3$, $B = 10^5 \mu$, $X^0(0) = 9 \times 10^4$, $X^1(0) = 10^4$, $Y_0^0(0) = 0.9$, $Y_0^1(0) = 0.1$ and $Y_1^1(0) = Y_1^0(0) = 0$.
