# Supplementary material for: Modelling the Evolution and Spread of HIV Immune Escape Mutants
Source: PLoS Pathog. 2010 Nov 18;6(11):e1001196. doi: 10.1371/journal.ppat.1001196 (PMC2987822; doi:10.1371/journal.ppat.1001196)
Supplement: Figure S1 — Confidence intervals for escape and reversion rates inferred from the cross-sectional data (dataset 2). (0.04 MB PDF) [file ppat.1001196.s008.pdf]

A

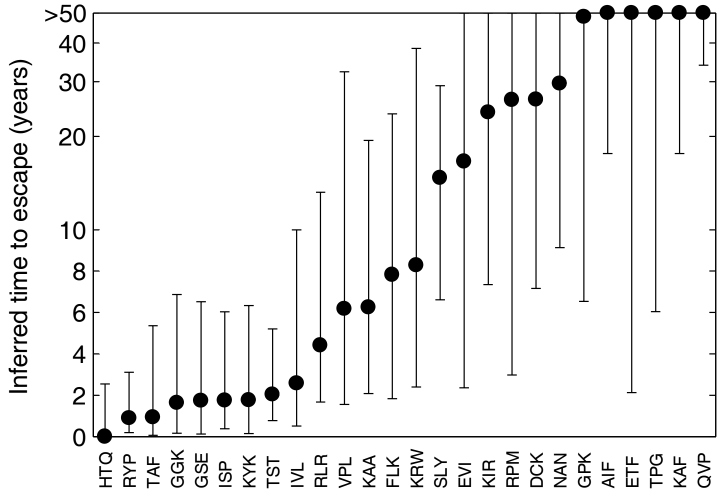

B

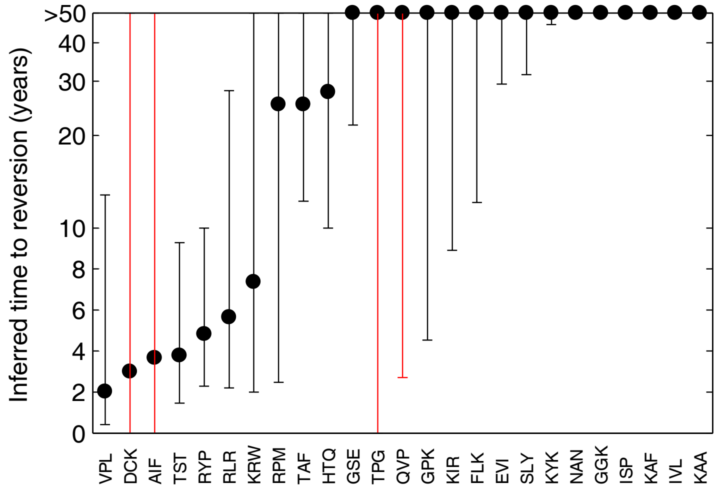

Figure S1. Confidence intervals for escape and reversion rates inferred from the cross-sectional data (dataset 2).

Best estimates (circles) and 95% confidence intervals (bars) for A) escape rates and B) reversion rates inferred from the cross-sectional data using the mathematical model. The best estimates are also presented in Figures 2A and 2B and Table S2. These confidence intervals account for sampling errors and were calculated by first calculating 95% confidence areas surrounding the escape prevalence data (as plotted in Figure 2B). These areas are calculated numerically assuming that the proportion of HLA-matched and mismatched hosts with escape are each drawn from independent binomial distributions. Confidence intervals surrounding the escape rates are estimated by calculating finding the maximum and minimum rates that correspond to escape prevalence data within the confidence areas. Notice that the size of the limits differs between epitopes. This is partly because of differences in the sample sizes and partly because differences in the underlying evolutionary rates. We highlight in red the four epitopes for which we have the least confidence in our inferred reversion rates. As plotted here, each of these 'best estimates' is >80% of the full length of the y-axis away from one of its 95% confidence limits. These epitopes are also highlighted in Figure 3B. Notice also that each of these epitopes has a very slow inferred escape rate. During the first few decades of an epidemic, slowly escaping mutants will have similarly low escape prevalences in the population, irrespective of the rates at which they revert. Our reversion rate estimates can therefore be sensitive to small changes in observed escape prevalences for epitopes that escape quickly. This is one example of why the confidence intervals are influenced by the underlying evolutionary rates. Estimates are provided for the epitopes in gag, RT and nef with previously defined escape mutants (Tables S1 and S2) excluding those for which cross-sectional sequence data (CGKEGHTAR, IPLTEEAEL and SRLAFHHVAR) or a defined HLA-restriction (AADTGHSNQ, HSNQVSQNYP) were not available. In addition, epitope ETF is excluded from B) because the reversion rate for this epitope was indeterminate. Thus, if we were to plot the confidence interval for ETF it would span the full range of the scale.
