# Supplementary material for: Modelling the Evolution and Spread of HIV Immune Escape Mutants
Source: PLoS Pathog. 2010 Nov 18;6(11):e1001196. doi: 10.1371/journal.ppat.1001196 (PMC2987822; doi:10.1371/journal.ppat.1001196)
Supplement: Figure S2 — Sensitivity analysis showing how the assumed model parameters affect our inferences from the cross-sectional data (c.f. Figure 3). (0.19 MB PDF) [file ppat.1001196.s009.pdf]

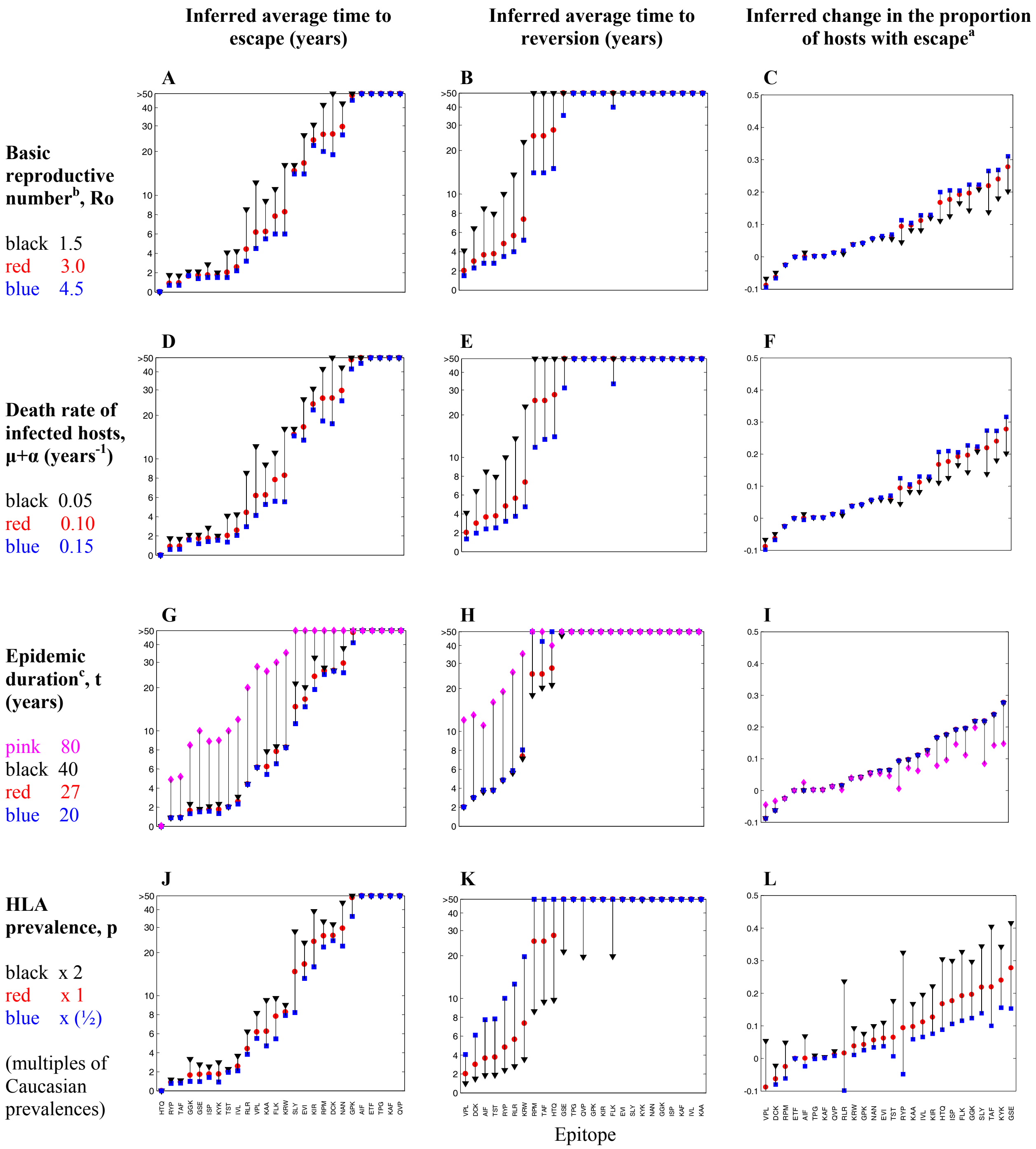

Figure S2. Sensitivity analysis showing how the assumed model parameters affect our inferences from the cross-sectional data (c.f. Figure 3).

We consider how changes to model parameters affect escape rates (column 1: A, D, G and J), reversion rates (column 2: B, E, H and K) and changes in escape prevalence in the population (column 3: C, F, I and L), inferred from the cross-sectional data (dataset 2). These results can be compared to the data plotted on the x-axes of Figure 3. They show that whilst our assumed model parameters affect the magnitude of our inferences from dataset 2, the rank order of the different epitopes remain largely unchanged for plausible alternative parameter estimates. Furthermore, changes in the magnitude of these estimates are relatively modest; we can still distinguish between rates measured in months, years or decades.

The following changes to model parameters are considered. Row 1 (A-C): the basic reproductive number (R_0). Row 2 (D-F): the death rate of infected hosts ($\mu+\alpha$), equal to the reciprocal of the life expectancy of infected hosts. Row 3 (G-I): the epidemic duration (t) and row 4 (J-L): the prevalence of the restricting HLAs (p) in the population. In each panel, the red circles show our 'best estimates', as presented in Figure 3. For these estimates we used the following parameters: $R_0=3$, $\mu+\alpha=0.1$ years^{-1}, t=27 years. HLA prevalences were estimated from Caucasians. For all other estimates, we kept all but one of these parameters fixed. The parameters that we changed are presented in the left hand column.

[a]In column 3 we have fixed the inferred escape and reversion rates to equal those shown by the red dots throughout this figure. Thus these estimates can be used to explore how changes in epidemic parameters between different epidemics can affect our inferences. [b]In this model, $R_0=\beta c/(\mu+\alpha)$, thus changing either the transmission coefficient (βc) or the death rate of infected hosts ($\mu+\alpha$) changes R_0. In row 1 the death rate is fixed, thus changes in R_0 are forced by changes in the transmission coefficient. [c]Note that the proportion of infected hosts at the start of the epidemic, Y(0)/X(0), can also affect the estimates by shifting how quickly the epidemic reaches saturation. We have not explored this effect explicitly. However, this effect can also be seen by choosing a large epidemic duration (80 years, pink markers) that pushes the epidemic dynamics to the equilibrium phase. For the remaining parameters explored in this figure, the epidemic dynamics are in the exponential growth phase.
