# Supplementary material for: Modelling the Evolution and Spread of HIV Immune Escape Mutants
Source: PLoS Pathog. 2010 Nov 18;6(11):e1001196. doi: 10.1371/journal.ppat.1001196 (PMC2987822; doi:10.1371/journal.ppat.1001196)
Supplement: Figure S3 — Observed changes in the population escape prevalence over approximately 20 years (dataset 1). (0.04 MB PDF) [file ppat.1001196.s010.pdf]

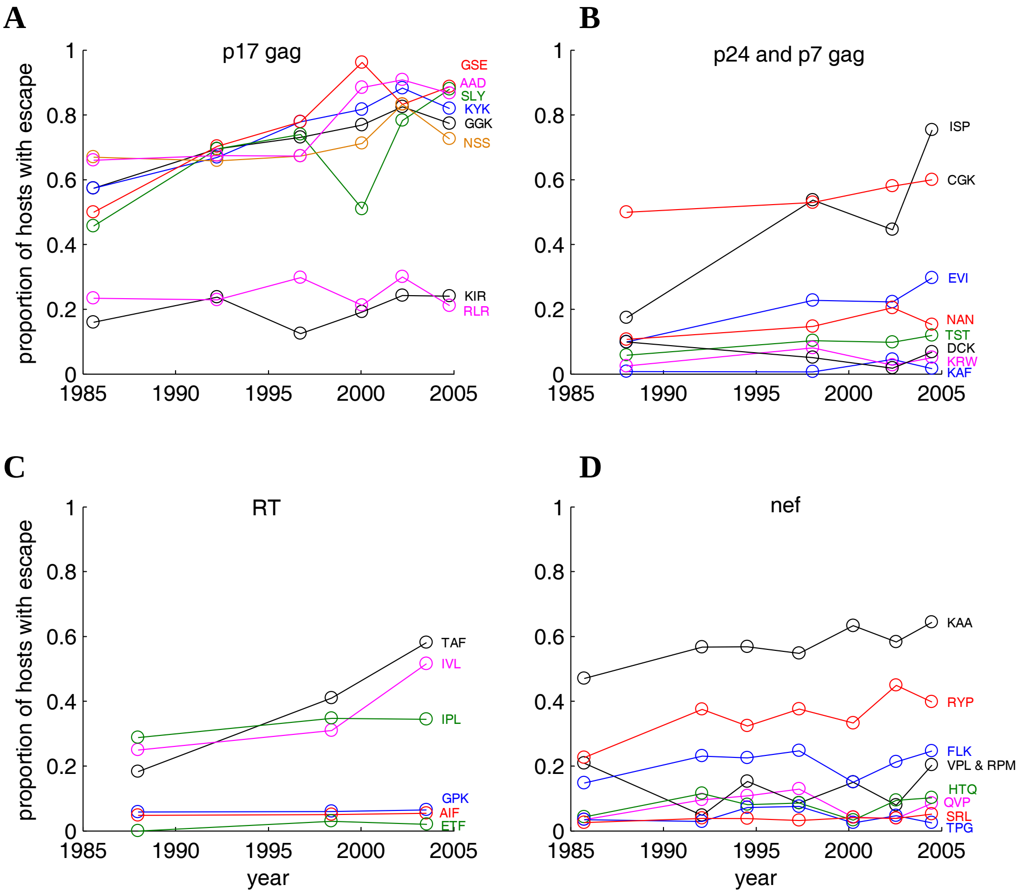

Figure S3. Observed changes in the population escape prevalence over approximately 20 years (dataset 1).

A)-D) Observed changes over time in the escape prevalence in 31 epitopes in gag (A and B), RT (C) and nef (D) with previously defined escape mutants (Table S1). In this analysis any mutation at a previously defined escape site is regarded as an escape mutant. These panels show that for some epitopes the prevalence of escape has remained relatively invariant over a 20 year period, whereas for other epitopes the prevalence of escape has markedly increased. The data used for these graphs were downloaded from the Los Alamos HIV Sequence Database (www.hiv.lanl.gov) using a search for dated B-clade sequences. The data from several years were then grouped together to ensure that each escape prevalence estimate is based upon approximately 100 individuals (range 93-136 individuals). No more than one sequence per individual was included from each date. These grouped estimates are plotted against the corresponding mean sample times of each grouped set of data.

Note that the data presented in this figure are used for calculating the observed changes in the escape prevalence at different epitopes provided in Fig. 3C. For each epitope the observed change is defined as the difference between the latest and earliest grouped estimate of the escape prevalence. For Fig. 3C the predicted change over the same period is estimated using the mathematical model parameterised by the escape and reversion rates inferred from the independent cross-sectional study (dataset 2, Table S2, columns 5 and 6). For those estimates the initial conditions of the model are defined so that the proportion of all hosts with escape is equal to the proportion observed at the earliest grouped sample time. Since the data downloaded from the Los Alamos database (dataset 1) are not HLA-typed, the initial conditions are also defined so that the ratio of HLA matched to HLA mismatched hosts with escape is equal to the ratio observed in the cross sectional data (Fig. 2B).
